# The effects of an object’s height and weight on force calibration and kinematics when post-stroke and healthy individuals reach and grasp

**DOI:** 10.1038/s41598-021-00036-9

**Published:** 2021-10-18

**Authors:** Ronit Feingold-Polak, Anna Yelkin, Shmil Edelman, Amir Shapiro, Shelly Levy-Tzedek

**Affiliations:** 1 Department of Physical Therapy, Recanati School for Community Health Professions, Ben-Gurion University of the Negev, Ben-Gurion Blvd, Beer-Sheva, Israel; 2 Beit Hadar Rehabilitation Center, Ashdod, Israel; 3 Department of Mechanical Engineering, Ben-Gurion University of the Negev, Beer-Sheva, Israel; 4 Zlotowski Center for Neuroscience, Ben-Gurion University of the Negev, Beer-Sheva, Israel; 5 Freiburg Institute for Advanced Studies (FRIAS), University of Freiburg, Freiburg, Germany

**Keywords:** Neuroscience, Health occupations, Neurology

## Abstract

Impairment in force regulation and motor control impedes the independence of individuals with stroke by limiting their ability to perform daily activities. There is, at present, incomplete information about how individuals with stroke regulate the application of force and control their movement when reaching, grasping, and lifting objects of different weights, located at different heights. In this study, we assess force regulation and kinematics when reaching, grasping, and lifting a cup of two different weights (empty and full), located at three different heights, in a total of 46 participants: 30 sub-acute stroke participants, and 16 healthy individuals. We found that the height of the reached target affects both force calibration and kinematics, while its weight affects only the force calibration when post-stroke and healthy individuals perform a reach-to-grasp task. There was no difference between the two groups in the mean and peak force values. The individuals with stroke had slower, jerkier, less efficient, and more variable movements compared to the control group. This difference was more pronounced with increasing stroke severity. With increasing stroke severity, post-stroke individuals demonstrated altered anticipation and preparation for lifting, which was evident for either cortical lesion side.

## Introduction

### Upper limb function following stroke

Cerebrovascular accidents (CVAs) are a leading cause of long-term disability worldwide^1^, leaving up to 75% of survivors with persistent upper limb (UL) sensorimotor deficits^2,3^. Impaired UL function post-stroke significantly impedes ability to perform activities of daily living (ADLs) such as reaching, picking up, and holding objects^4^. These deficits limit performance and social participation, negatively affecting quality of life^3,5–7^.

### Reach-to-grasp movement in healthy and post-stroke individuals

Reach-to-grasp (RTG) movements are a primary means of interacting with the environment, allowing people to obtain and manipulate objects around them^8^. RTG movement entails both the transport component, which is the change in position of the hand over time, and the grasp component^9^. Both are synchronized such that the hand opens and closes, in coordination with hand movements when grasping objects^10^. RTG movements require precise application of grip forces^11^, e.g., when we move our arm while holding an object between our fingers, we unconsciously increase the grip forces to prevent the object from slipping^12^ or sliding^13^. Skilled grip force relies on prediction and sensory feedback^14^, such that during a grip-lift task, healthy individuals are able to rapidly establish an association between an arbitrary sensory cue with a given weight and scale grip force precisely to the actual weight^15^. Movement trajectories by healthy adults involve more than one joint, tend to be straight, smooth, and have bell-shaped velocity profiles^16–18^.

In contrast, goal-directed RTG movements post-stroke are characterized by slowness, spatial and temporal discontinuity, and abnormal patterns of muscle activation and joint synergies^5,17,19^. Persons with stroke tend to use a stereotypical shoulder elevation movement as well as anterior trunk displacement up to 4.5 times more than normal^20,21^ to compensate for lack of shoulder and elbow movement when reaching their arm^22^. These compensatory behaviors were observed in pointing tasks^17^, reaching for different targets in the workspace^23^, obstacle avoidance^3^, bimanual reach at different heights^20^, and functional tasks such as reaching for a cup and drinking^24–26^.

### Force regulation post stroke

Deficits in force efficiency post-stroke were reported to be greater in the *grasp* than in the *reach* phase^27^. Moreover, as opposed to the reaching movement, grasp efficiency did not recover over time and was found to be the greatest remaining deficit post stroke^27^.

Previous studies found that the maximal grip force of stroke survivors is reduced by 43–75% compared to that of control participants^28–30^, and yet they use inadequately high grip forces when grasping objects due to abnormal force regulation^11^. Their increased force variability can reduce the accuracy of force production^29,30^. However, there is evidence that persons in the chronic phase of stroke preserve the ability to modulate grip force within their limited force range^29^. Blennerhassett et al.^31^ identified two principle components in the grip-force regulation of persons with stroke: “Pre-Lift Delay” and “Grip-Force Dyscontrol”. Pre-Lift Delay describes the use of more time and larger amounts of grip force at the onset of the task. Grip-Force Dyscontrol describes the use of excessive and more unsteady force across the entire grip task.

### RTG to different heights post-stroke

It has been demonstrated that our goal when reaching to an object (e.g., do I reach for a cup in order to hand it to someone else, or in order to pour water into it) affects the way we perform the movement^32–35^. Therefore, if we wish to study how people after stroke reach to grasp an object and how they regulate their force production when they perform ADL, we must study their movement when they are engaged in a *functional* and *purposeful everyday task*, using an everyday object as part of the measurement apparatus, and designing the environment accordingly. However, when reviewing the literature, we encountered three limitations in the study of RTG movement. First, the majority of the studies on reaching tasks of persons with stroke do not include a grasp phase^8,9^, although movements in daily activities commonly involve not only a reaching component but also grasping and lifting of objects^24,27^, affecting people's motor planning and execution^32,36^.

Second, post-stroke RTG movement has been studied widely, yet there is limited information regarding the kinematics and the dynamics of force regulation of RTG movements during a function-oriented task and the ability of the paretic arm to adjust to different heights and weights. Most research examined reach movements at standard table height^20,37–39^ though ADLs performance requires reach in three-dimensional space, not just at one height on a transverse plane^20^. Few studies measured kinematic deficits post-stroke at different heights^40–42^. Higher endpoint error, shoulder flexion, and abduction range of motion were evident for movements to higher targets^40^. Objects at higher heights required greater compensatory muscle recruitment of proximal UL muscles on the paretic side than the non-paretic side^42^. Post-stroke individuals had difficulty using combinations of muscles selectively to stabilize the trunk’s trajectory; this was reported to be more evident when reaching upward than downward, perhaps due to gravity^41^.

Third, while the negative impact of poor functional grasp (e.g., of a bottle or a cup) and lack of a functional reach task on ADLs is well described in the literature^24–26,43^, grip forces are often not studied in the context of an everyday-task (e.g., when gripping a cup), but rather using tools such as the dynamometer^28,44^ or the "Pinch, Grip, Lift and Hold" apparatus^31,45,46^. Reach tasks included virtual targets^38,39,47^ or grasp of non-functional targets, such as moving a polystyrene ball^37^, grasping a sensor^48^, and touching a rod attached to a circular base^40^. Few studies^24–26,43^ used a cup as a target object, however, there was no variability in the cup's height or weight. Several reach-movement studies in virtual reality (VR) with stroke participants have been conducted to date^3,47,49^ allowing for a variety of tasks to be safely performed. Yet upper limb kinematics in VR differ from physical environments^47,49^ as the technology still lacks the ability to provide haptic feedback to users^49^. In summary, reaching difficulties are common post stroke and correlate strongly with general impairment^50^. For this reason, coordination of RTG is a primary rehabilitation goal^9^. RTG post-stroke has been widely studied, yet to date there is incomplete information about RTG performance at different heights and weights of the target during functional task performance and the correlation between the kinematics and the kinetics during such a task. Characterizing the differences between stroke and healthy individuals when performing an ecological RTG movement can serve as the basis for developing appropriate therapeutic interventions^14^.

The aim of this study was thus to evaluate movement quality, efficacy, and the force regulation of the affected UL of sub-acute post-stroke individuals during a functional RTG task at different heights and weights compared to healthy individuals. We hypothesized that, compared to healthy individuals: (1) the movement of individuals with stroke will be slower, less accurate, less smooth with less efficient force regulation; (2) impaired force regulation of post-stroke individuals would be more evident when grasping the heavier cup, placed at the highest location; (3) individuals with stroke will show a decreased efficacy of movement and excessive compensatory joint movements when they reach to grasp a heavier cup, located higher; and (4) individuals with a severe impairment level (FMA < 35) will show a more pronounced impairment in force regulation and kinematic measures than individuals with mild or moderate impairment (FMA ≥ 35).

## Methods

### Participants

A total of 46 participants took part in this cross-sectional study. Thirty hospitalized post-stroke participants were recruited from the in-patient population of “Beit-Hadar” Rehabilitation-Center (16 males, 14 females, mean age 70.3 ± 9.3 years, mean time post-stroke 46 ± 19.9 days; 23 had middle cerebral artery (MCA) stroke, three had posterior cerebral artery (PCA), two had anterior cerebral artery (ACA), one suffered a stroke in the cerebellum, and one suffered a stroke in the pons; See Table S1 in Supplementary Materials). In addition, a convenience sample of 16 age-matched (t = 0.38, *p* = 0.70) healthy-control participants were recruited from the community (5 males, 11 females, mean age 69.1 ± 11.5 years; See Supplementary Materials S1).

Post-stroke participants with the following characteristics were included: (a) first unilateral-stroke^24^, (b) age 50–85 years^48^, (c) Mini-Mental State Examination score ≥ 24/30^51^, (d) Fugl-Meyer Upper-Extremity assessment (FMA) score 16–66: a validated four-level classification of the FMA was used in order to include only post-stroke participants who have at least some movement ability in the affected arm^52^, (e) no excessive pain in the affected arm, defined as ≤ 4 on the Visual Analogue Scale, (f) a score of ≤ 2 on the Modified Ashworth Scale^53^, (g) Brunnstrom stages of motor recovery ≥ 3/7^8^, and (h) ability of the participant to sit independently without external support.

Post-stroke individuals with additional neurological or musculoskeletal problems, with severe vision or sensory deficits affecting upper limb motor ability, or with aphasia affecting understanding of simple instructions, were excluded^54^.

All participants gave their written informed consent, in accordance with the requirements of the Barzilai Hospital Helsinki Ethical-Committee, which approved the experimental protocol (0014–17-BRZ).

### Experimental procedure

#### Evaluation procedure

All participants were individually examined by a physical therapist. Evaluations of participants with stroke were performed in two sessions, each lasting one hour, on consecutive days, in order to avoid fatigue. The clinical tests were performed on day 1, and the force and kinematic measurements, on day 2. Evaluation of the control group was performed in one session, lasting approximately 45 min.

#### Experimental set-up

**Figure 1:**
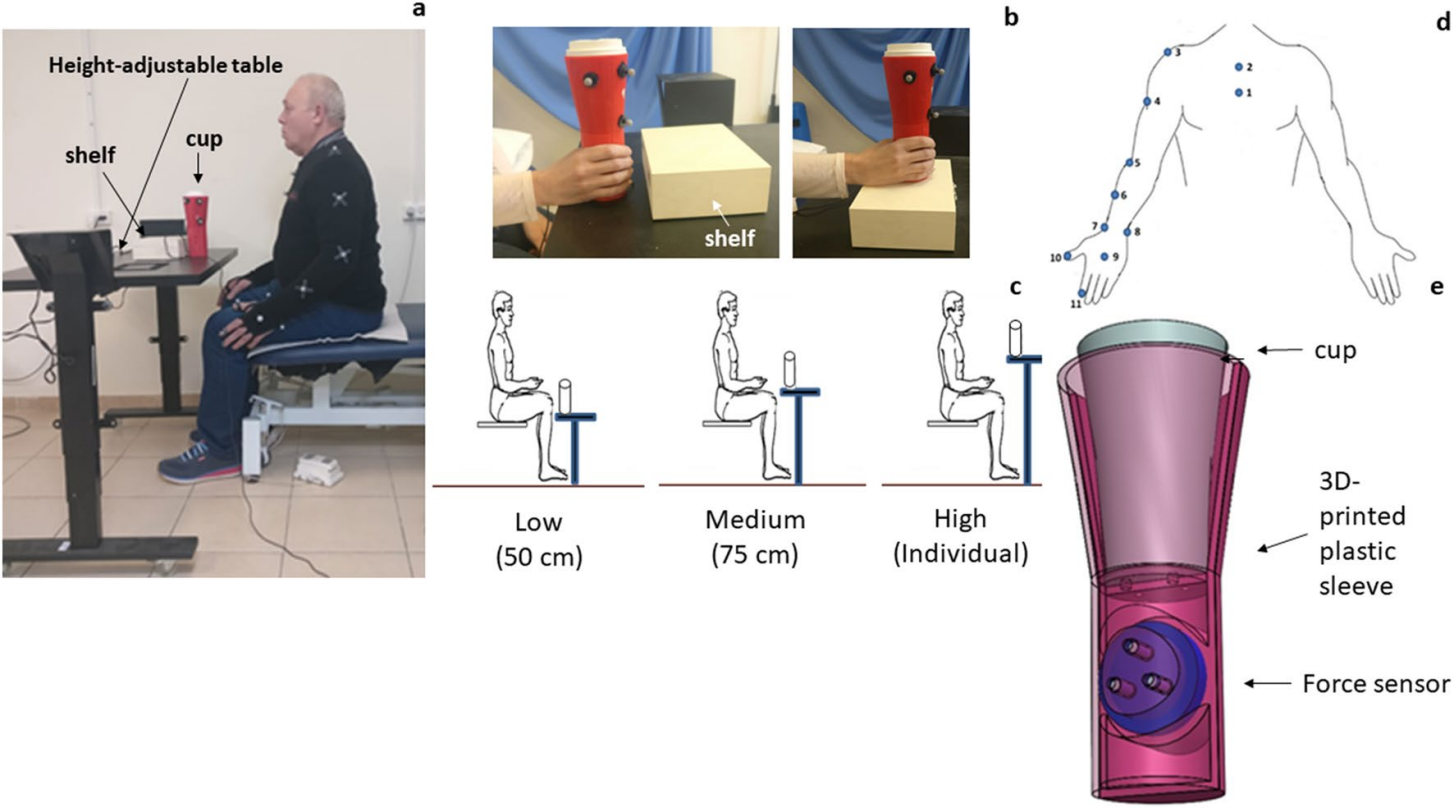
The experimental setup. (**a**) A control participant sitting in front of the table adjusted to medium height. The cup (red) is at its starting position and the target shelf (white) is behind the cup; (**b**) The initial (*left*) and the final (*right*) position of the cup during the task; (**c**) A schematic of the three target heights; (**d**) Position of the markers. Position of the markers are illustrated on the right upper extremity: (1 + 2) Sternum, (3) Shoulder (lateral acromion of the scapula), (4) Proximal humerus, (5) Lateral epicondyle, (6) Middle forearm, (7) Radial styloid process, (8) Ulnar styloid process, (9) Dorsal side of the palm (wrist), (10) Thumb fingertip, and (11) Index fingertip; (**e**) An illustration of the custom-built cup with the embedded 3D force sensor in it. The participant provided informed consent for publication of his identity revealing images.

The experimental design was already described elsewhere^55^. In brief, the measurements were performed while the participants sat, without trunk support, in front of a height-adjustable table (for the medium and higher height) (see Fig. 1a) and next to the table (for the lower height). Participants were instructed to do the following, once they heard a "beep" sound: to reach their hand at a self-selected speed toward a cup located on the table, then lift the cup and place it on top of a 5 cm-high block positioned on the table (see Fig. 1b). The cup was horizontally aligned with their reaching arm, and placed in one of three different heights, each of which is relevant for everyday tasks: (a) *low*: the height of the wrist when the hand is extended downwards when the person is seated (50 cm above the ground); (b) *medium*: the height of a standard table (75 cm above the ground); and (c) *high*: the height of the shoulder (adjusted individually to each participant; range 86-104 cm, average 92.3 cm) (see Fig. 1c). The cup was placed at an arm's distance, measured from the lateral acromion to the radial styloid process, to avoid excessive trunk movement during reaching movement. The participants were instructed to reach, grasp, and lift the cup in one continuous movement, and to avoid bending the trunk as much as possible during reaching movement, but the movement of the trunk was not restrained.

In order to emphasize the everyday functionality of the task, in addition to the height variability, reach and grasp movements were executed using two different weights of cups: an empty cup (273 g) and a cup filled with water (443 g). Participants were told whether the cup was empty or full, and the cup was capped to prevent spilling. The reach movement was executed by the impaired arm of participants with stroke, which may be their dominant or their non-dominant arm. Therefore, we verified that the percent of control participants reaching with their dominant arm was similar to the percent of participants with stroke who reached their dominant arm^9,11^. The starting position for the *low* height was with the arm held vertically at the side of the body, and for the *medium* and *high* heights, it was with the hand placed on the ipsilateral thigh with palm facing down. Every combination of height and weight was evaluated up to three times, depending on the participant's ability. That is, while the maximal total number of reaching trials was 18 (3 heights × 2 weights × 3 repetitions), some stroke participants were not able to complete all the trials, due to arm weakness or fatigue. For each participant, the order of the table heights (low, medium, high) was randomly set using a computer program. Once the height was chosen, the order of the cup weights (empty first or full first) for this height was also randomly set using a computer program.

### Equipment

#### Kinematic measurements

Position of the UL joints was recorded using the V120:Trio portable motion-capture system (OptiTrack, NaturalPoint, Inc., USA), composed of three cameras (120 Hz). No calibration is required. Eleven reflective markers were placed on the upper body as follows (the numbers in brackets correspond to the numbers in Fig. 1d): two on the sternum (1 + 2), vertically aligned (to measure trunk motion), and one on each of the following anatomical landmarks: shoulder-lateral portion of the acromion (to reflect the scapular motion) (3), proximal humerus (4), lateral epicondyle of the elbow (5), the middle forearm (6), radial (7) and ulnar (8) styloid processes, the dorsal side of the palm at the axis along the middle of the third metacarpal bone (to reflect the wrist motion) (9), thumb (10) and index fingers (11). Two additional stationary markers were placed vertically on the wall as reference points, and three additional markers were placed on the cup and were defined by the system as a rigid body, so that cup location was tracked as well.

#### Kinetic measurements

We used a custom-built 3D-printed cup. The diameter at the gripped (bottom) portion of the cup was 6.5 cm; the height of the cup was 20.3 cm. Grip forces were measured using a 3D force sensor (Nano25-E Transducer, ATI Industrial Automation, INC) embedded in the cup (see Fig. 1e). The data-sampling speed of the force sensor was 100 Hz.

#### Clinical evaluations

Participants from the stroke group were evaluated using two clinical measurements. The FMA was used as an analog to the Body Functions & Structures component of the ICF model^56^. The participants with stroke were grouped into three groups, based on the severity level of their impairment, as measured by FMA scores (16–34 = Moderate-severe impairment, 35–53 = Moderate impairment, and 54–66 = Mild impairment)^52^. The Chedoke Arm and Hand Activity Inventory (CAHAI) 7-item version was used as an analog to the Activity component of the model^57^.

#### Outcome measures

Each movement was segmented into three phases: Reach, Grasp, and Lift. We analyzed the data separately for each of these three movement phases: Reach phase (from starting position until grasp of the cup); Grasp phase; and Lift phase (from grasp until placement of the cup on the shelf). The phases were determined using the following four time points: the time of movement initiation (T1), the time at which the cup was grasped (T2), the time at which the cup was lifted (T3), and the end of the movement, once the cup was placed on the shelf (T4)^55^. For the details on how T1–T4 were calculated, see Supplementary Materials.

#### Kinetic outcome measures

The data of the force sensor were filtered using a 10 Hz Butterworth filter; we then calculated the following outcome measures:

1. *Mean force:* The mean grip force applied to the cup in Newtons^11,30^. Calculated for the Grasp phase (T2–T3) and the Lift phase (T3-T4).
2. *Peak force:* The maximal force applied to the cup in Newtons^24^. Calculated for the Grasp phase (T2–T3) and the Lift phase (T3-T4).
3. *Time-to-peak-force:* The time it took the participant to reach the peak force^58^. Calculated in seconds from the time the reaching movement started (T1).
4. *Efficiency*: The efficiency of the applied force during the task was determined using three measures:
  - *Force-to-time ratio*: The mean force during a phase, in Newtons, divided by the duration of that phase in seconds. Calculated for the Grasp phase (T2–T3) and the Lift phase (T3–T4).
    For example:
    $$Force{\text{-}}to{\text{-}}time\;ratio\;GRASP = \frac{{{\text{Mean}}\;{\text{force }}\left( {{\text{T}}2 - {\text{T}}3} \right)}}{{{\text{Time }}\left( {{\text{T}}2 - {\text{T}}3} \right)}}$$
  - *Variability of force:* To measure variability of forces, representing the pre-lift delay and the grip force dys-control^45^, two variables were calculated:
    (1) Coefficient of variation (CV): CV expresses variability relative to the mean force level^29^. It was calculated by dividing the standard deviation of the force values during the Grasp and Lift phases by the mean force applied during that period (T2–T4)^29,30,59,60^:

$$ CV = \frac{{Standard\;deviation\;of\;force}}{{Mean\;grip\;force}} $$

- (2) Number of force peaks (NFP): The number of force peaks performed during the movement. We determined the NFP as follows: (i) We calculated F_T_, the value of 5% from the maximal force exerted during the trial; (ii) We identified all local maxima (peaks) and local minima in that trial's force trace; (iii) Only peaks which had a value greater than [F_T_ + the preceding local minimum value] were counted as force peaks. The maximal peak can be either in the *Grasp* phase or in the *Lift* phase; Thus, for some of the participants, there are cases when there is no local peak in one of the phases (Grasp/Lift).

#### Kinematic data analysis

Kinematic data were filtered using the Butterworth filter with a cutoff of 20Hz^60–63^, and then used to calculate the following kinematic outcome measures:

1. *Movement duration and velocity:* Movement duration was calculated for the entire task, between movement onset (T1) and end (T4), as well as separately for each phase. Mean and peak velocities were determined from tangential velocity traces of the wrist marker. Time to peak velocity (TTPV) was also calculated.
2. *Joint Angles:* Maximum and minimum angle values, and the difference between them (D_angle__=_ Maximal_Angle − Minimal_Angle), were calculated for all phases of the task. UL joint angles were calculated as detailed in the Supplementary Materials.
  *Trunk Displacement* (TD)^20,26,64,65^ was calculated as the distance, in cm, of the z component of the sternum marker (marker 2 in Fig. 1d) from resting position till the end position of the task (T4).
3. *Trajectory Smoothness:* Smoothness can be assessed using several measurements^66,67^. We quantified the smoothness by the normalized jerk (NJ) as described by Buma, et al.^68^. The jerk was normalized by the movement duration, and by the distance travelled^68^. Specifically, NJ was calculated as follows:
  $$NJ = \sqrt {\frac{1}{2}\int\limits_{{t_{{{\text{start}}}} }}^{{t_{{{\text{end}}}} }} {jerk^{2} \left( t \right){\text{d}}t} {\text{*MD}}^{5} /{\text{L}}^{2} }$$
  where NJ represents the normalized jerk of the movement; *t*_start_ represents the time the movement started; *t*_end_ represents the time at which the movement ended; jerk represents the third time derivative of the position with respect to time; MD represents the movement duration and L represents the distance traveled between the start and end of the movement. This normalization means that NJ is mathematically independent of the movement duration and the distance travelled^67,68^. NJ values were log-transformed, to meet assumptions of normality^68^.
4. *Trajectory straightness*, defined as the index of curvature (IC) or the ratio between the length of the trajectory and the length of a straight line between the initial and final hand locations^3,23,49,69^. An IC value of 1 represents a straight hand path, whereas an IC value greater than 1 represents a curved path or multiple attempts to reach for the cup.

### Statistical analysis

Data were analyzed using SPSS (Statistical Packages for Social Sciences, 26.0) and a custom-written script in MATLAB software (Mathworks, MA, v.R2018b). We used a linear mixed model (LMM) to analyze the results of the repeated measures^26^ across the two groups, with a between-subject factor (*group*: stroke/control) and one within-subject factor (*congruence*, (indicating whether the hand performing the RTG movement was the dominant one): yes/ no) and the interaction between these factors. For each dependent force variable, a model that included the fixed effects of group and congruence was evaluated. Only participants with a hemispheric stroke were calculated in the congruent-side analysis. In order to analyze the results of the repeated measures^26^ across the three severity levels within the stroke group, and how they compare to the control group, we used another LMM, with a between-subject factor (*group*: mild/moderate/moderate-severe/control) and two within-subject factors (*height*: low/medium/high; and *weight*: empty/full) and the interaction between these factors. For each dependent force variable, a model that included the fixed effects of FMA level, height, and weight was evaluated. The models were adjusted for trial order. For all post-hoc tests, the *p* values were adjusted using the Bonferroni correction for multiple comparisons; significance level was set at *p-value* < 0.05.

We used the Spearman rho correlation test to test the relationship between the kinematic and kinetic measures (values averaged over each participant's 18 trials).

## Results

### Clinical assessments

The mean score of the FMA was 46.5 (± 9.8), ranging from 30–62 points, out of 66. According to the FMA level classification^52^, 33% of the participants with stroke (n = 10) were classified as having a *mild* motor impairment of the UE, 50% (n = 15) had a *moderate* impairment and 17% (n = 5) had a *moderate-severe* impairment. The mean score of the CAHAI-7 was 34.9 (± 8.9), ranging between 16–47 points. There was no difference in the distribution of FMA score between the patients who used their dominant hand and those that used their non-dominant hand to perform the task (t_26_ = -0.16, *p* = 0.874).

We analyzed a total of 828 reach-to-grasp movements performed by the 46 participants. The results of their analysis are detailed below.

### Force variables during grasp and lift phases

Tables 1 and 2 summarize the results of the force variables of the stroke and the control groups during the Grasp and the Lift phases.

**Table 1:** Mean values of force variables: group and lateralization effects.

	Group						Congruent									
	Grasp		F	Lift		F	Grasp				F	Lift				F
	Stroke (n = 30)	Control (n = 16)		Stroke (n = 30)	Control (n = 16)		Stroke		Control			Stroke		Control		
							Yes	No	Yes	No		Yes	No	Yes	No	
Mean force (N)	4.4 (1.2)	4.8 (1.5)	F_1,495_ = 3.64	8.5 (5.1)	8.9 (2.8)	F_1,719_ = 2.69	**3.3 (0.2)**	**5.6 (0.2)**	4.7 (0.2)	4.6 (0.2)	**F**_**1,695**_ = 57.07***	**5.9 (0.3)**	**10.8 (0.3)**	8.4 (0.3)	7.9 (0.4)	**F**_**1,719**_ = 65.15***
Peak force (N)	11.1 (0.3)	11.4 (0.4)	F_1,677_ = 0.31	13.4 (0.3)	13.4 (0.5)	F_1,719_ = 0.002	**8.1 (0.4)**	**14.1 (0.4)**	11.0 (0.4)	11.1 (0.5)	**F**_**1,677**_ = 64.44***	**10.0 (0.5)**	**16.9 (0.5)**	12.7 (0.4)	12.2 (0.5)	**F**_**1,720**_ = 55.68***
Time-to-peak (sec)	**6.3 (0.2)**	**3.0 (0.5)**	**F**_**1,320**_ = 40.41***	**8.9 (0.4)**	**3.2 (0.1)**	**F**_**1,487**_ = 123.79***	**5.0 (0.3)**	**7.7 (0.4)**	2.2 (0.1)	2.1 (0.2)	**F**_**1,347**_ = 26.39***	8.4 (0.5)	9.3 (0.6)	**3.1 (0.1)**	**3.4 (0.1)**	F_1,484_ = 2.19
CV	0.8 (0.01)	0.8 (0.02)	F_1,755_ = 0.23	0.4 (0.6)	0.4 (0.4)	F_1,763_ = 6.47	**0.7 (0.02)**	**0.8 (0.02)**	**0.7 (0.01)**	**0.8 (0.02)**	**F**_**1,755**_ = 17.43***	0.4 (0.01)	0.4 (0.01)	0.4 (0.01)	0.4 (0.01)	F_1,763_ = 2.84
Force-to-time ratio (N/sec)	**4.7 (0.2)**	**10.7 (0.3)**	**F**_**1,736**_ = 309.91***	**3.0 (0.1)**	**5.6 (0.1)**	**F**_**1,712**_ = 196.75***	**3.9 (0.3)**	**5.6 (0.3)**	10.3 (0.4)	10.7 (0.5)	**F**_**1,737**_ = 12.83***	**2.3 (0.1)**	**3.8 (0.1)**	5.7 (0.2)	5.2 (0.3)	**F**_**1,712**_ = 19.87***
NFP	**1.5 (0.1)**	**0.2 (0.1)**	**F**_**1,707**_ = 89.86***	**1.9 (0.2)**	**0.9 (0.2)**	**F**_**1,697**_ = 27.53***	**2.0 (0.1)**	**1.0 (0.1)**	**0.3 (0.04)**	**0.1 (0.05)**	**F**_**1,707**_ = 26.24***	**2.5 (0.2)**	**1.4 (0.2)**	0.9 (0.1)	0.8 (0.1)	**F**_**1,697**_ = 14.57***

Summary of the results. Group: lists the mean values (SE); Congruent: lists the mean values (SE), Yes—the examined hand was the dominant one; No—the examined hand was the non-dominant one. Bolded values denote a significant difference between the groups. Asterisks denote the *p* value: *p < 0.05, **p < 0.01, ***p < 0.001.

*N* Newtons, *sec* Seconds, *CV* Coefficient of variance, *NFP* Number of force peaks.

**Table 2:** Mean values of force variables: effect of height, weight, and severity.

	Height		Weight		Severity	
	Grasp	Lift	Grasp	Lift	Grasp	Lift
	F	F	F	F	F	F
Mean force (N)	**F**_**2,465**_ = 19.122***	**F**_**2,524**_ = 5.762**	**F**_**1,723**_ = 48.556***	**F**_**1,760**_ = 30.184***	**F**_**3,723**_ = 13.929***	**F**_**3,760**_ = 11.727***
Peak force (N)	**F**_**2,409**_ = 18.933***	**F**_**2,546**_ = 7.08**	**F**_**1,708**_ = 44.99***	**F**_**1,755**_ = 28.03***	**F**_**3,708**_ = 4.94**	**F**_**3,755**_ = 4.31**
Time-to-peak (sec)	F_2,179_ = 0.26	F_2,318_ = 1.48	F_1,260_ = 0.69	F_1,483_ = 0.16	**F**_**3,262**_ = 28.82***	**F**_**3,477**_ = 75.96***
CV	**F**_**2,544**_ = 3.8*	F_2,546_ = 0.03	F_1,767_ = 1.93	F_1,786_ = 0.33	F_3,767_ = 0.155	**F**_**3,786**_ = 34.89***
Force-to-time ratio (N/sec)	**F**_**2,504**_ = 4.13*	**F**_**2,490**_ = 4.28*	**F**_**1,781**_ = 8.52*	**F**_**1,741**_ = 18.57***	**F**_**3,781**_ = 133***	**F**_**3,741**_ = 96.41***
NFP	**F**_**2,520**_ = 0.015*	**F**_**2,536**_ = 5.59**	F_1,742_ = 0.054	F_1,768_ = 3.08	**F**_**3,742**_ = 43.69***	**F**_**3,768**_ = 42.81***

Summary of the forces results. Bolded values denote a significant difference between the groups. Height, Weight, Severity: list the F value for the differences across this parameter; Asterisks denote the *p* value: *p < 0.05, **p < 0.01, ***p < 0.001.

*N* Newtons, *sec* Seconds, *CV* Coefficient of variance, *NFP* Number of force peaks.

### Mean force

**Figure 2:**
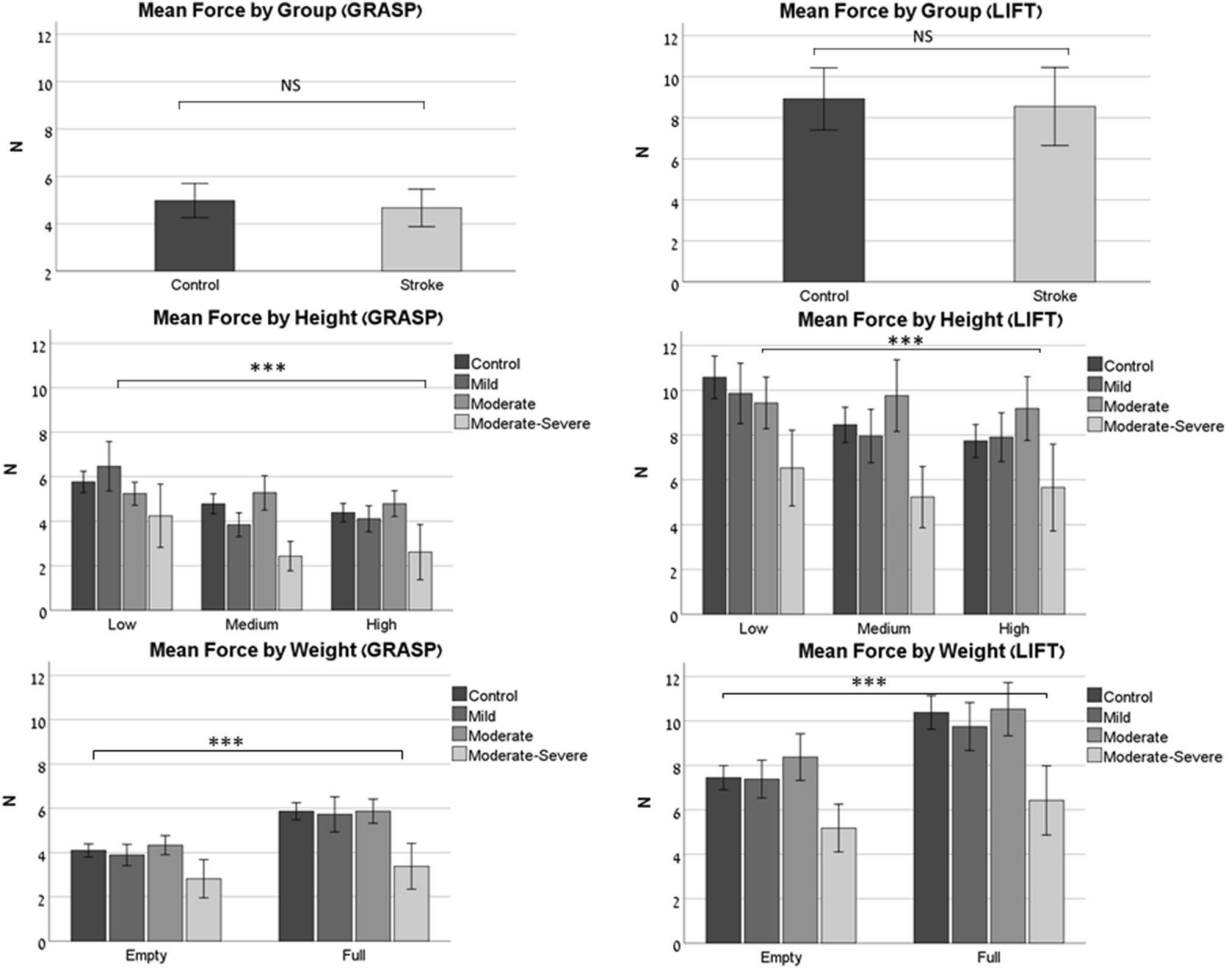
Mean force (N). Mean-force values for the Grasp phase (*left*), and the Lift phase (*right*). The top row shows the results by group (control/stroke). The middle row shows the results by height (low/medium/high), and by sub-group (control/mild impairment/moderate impairment/moderate-severe impairment). The bottom row shows the results by weight (empty/full cup, corresponding to light/heavy weight), and by sub-group. Asterisks denote the *p* value: *p ≤ 0.05, **p ≤ 0.01, ***p ≤ 0.001. Abbreviations: N-Newtons.

The results of the mean forces are presented in Fig. 2.

Group effect: There was no significant difference in the mean forces applied by the two groups (*Grasp*: F_1,726_ = 0.746, *p* = 0.39; *Lift*: F_1,758_ = 0.013, *p* = 0.91). There was no difference in mean force between hands in the control group. However, in the stroke group, the participants who used their non-dominant hand (this was the affected side for them) exerted a significantly higher mean force in both phases (*Grasp*: F_1,432_ = 90.41, *p* < 0.001; *Lift*: F_1,449_ = 107.83, *p* < 0.001) than those who used their dominant one.

Each of the factors (height/weight/stroke severity) had a significant influence on the mean force during the *Grasp* (Height: F_2,465_ = 19.122, *p* < 0.001; Weight: F_1,723_ = 48.556, *p* < 0.001; Stroke severity: F_3,723_ = 13.929, *p* < 0.001) and the *Lift* (Height: F_2,524_ = 5.762, *p* = 0.003; Weight: F_1,760_ = 30.184, *p* < 0.001; Stroke severity: F_3,760_ = 11.727, *p* < 0.001). The moderate-severe group produced significantly less force than the two other stroke groups and the control group during both the *Grasp* and *Lift* phases (*p* < 0.001).

Height effect: *Grasp:* Participants in both groups produced higher mean forces when grasping the cup at the low height compared to the medium and the higher heights (*p* < 0.001) with no significant difference between the two groups. *Lift:* Participants in the control group produced significantly higher mean forces when lifting the cup at the lower height compared to the higher height (*p* < 0.001), this difference was not present in the stroke group.

Weight effect: *Grasp & Lift*: Participants in both groups produced higher mean forces when grasping and lifting a full cup, compared to an empty one (*p* < 0.001), with no significant difference between the two groups.

### Peak force

**Figure 3:**
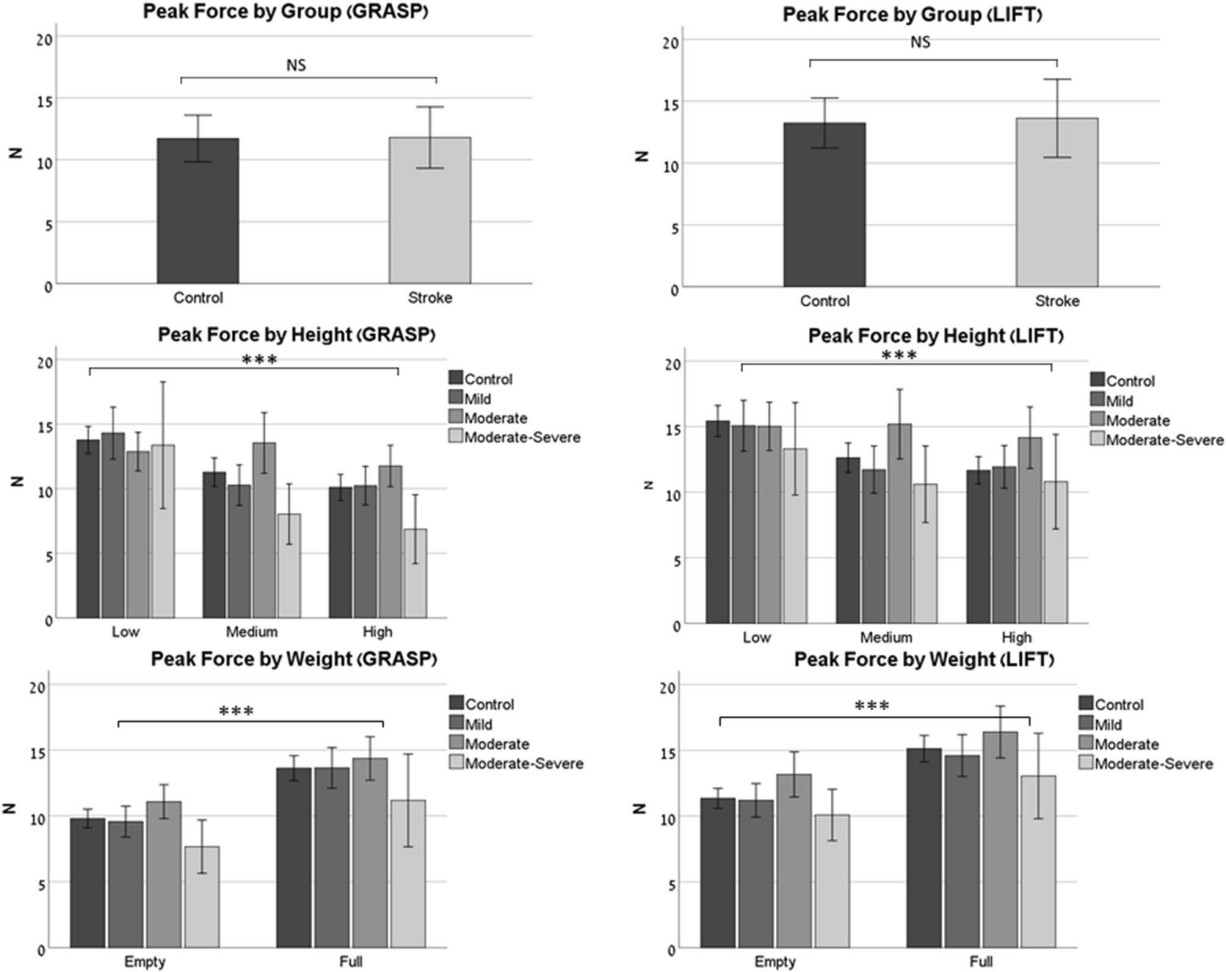
Peak force (N). Peak-force values for the Grasp phase (*left*), and the Lift phase (*right*). The top row shows the results by group (control/stroke). The middle row shows the results by height (low/medium/high), and by sub-group (control/mild impairment/moderate impairment/moderate-severe impairment). The bottom row shows the results by weight (empty/full cup, corresponding to light/heavy weight), and by sub-group. Asterisks denote the *p*-value: *p ≤ 0.05, **p ≤ 0.01, ***p ≤ 0.001. Abbreviations: N-Newtons.

The results of the peak force are presented in Fig. 3.

Group effect: There was no difference in the peak forces between the groups (*Grasp*: F_1,718_ = 0.15, *p* = 0.695 *Lift*: F_1,758_ = 0.002, *p* = 0.966). In the stroke group, the peak forces were higher when the tested (affected) hand was the non-dominant one (*Grasp*: F_1,431_ = 87.2, *p* < 0.001; *Lift*: F_1,459_ = 80.94, *p* < 0.001). This difference was not demonstrated among the control. All three factors (height/weight/stroke severity) had a significant influence on peak-force during both *Grasp* (Height: F_2,409_ = 18.933, *p* < 0.001; Weight: F_1,708_ = 44.99, *p* < 0.001; Stroke severity: F_3,708_ = 4.94, *p* = 0.002) and *Lift* (Height: F_2,546_ = 7.08, *p* < 0.001; Weight: F_1,755_ = 28.03, *p* < 0.001; Stroke severity: F_3,755_ = 4.31, *p* = 0.005): higher peak forces at the lower height and when the cup was full (F_2,499_ = 3.59, *p* = 0.03).

### Efficiency measures

#### Force-to-time ratio (FTR)

**Figure 4:**
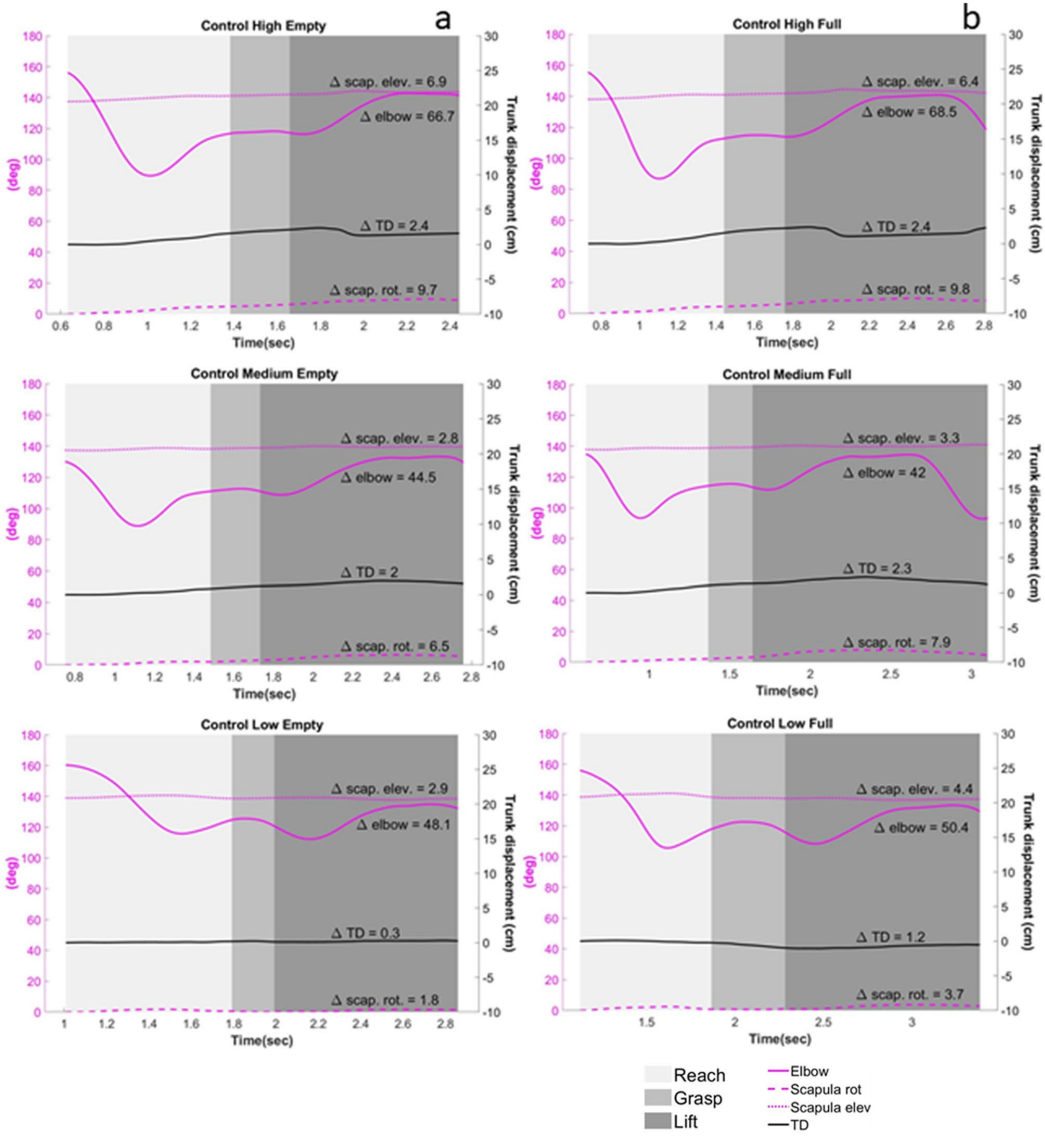

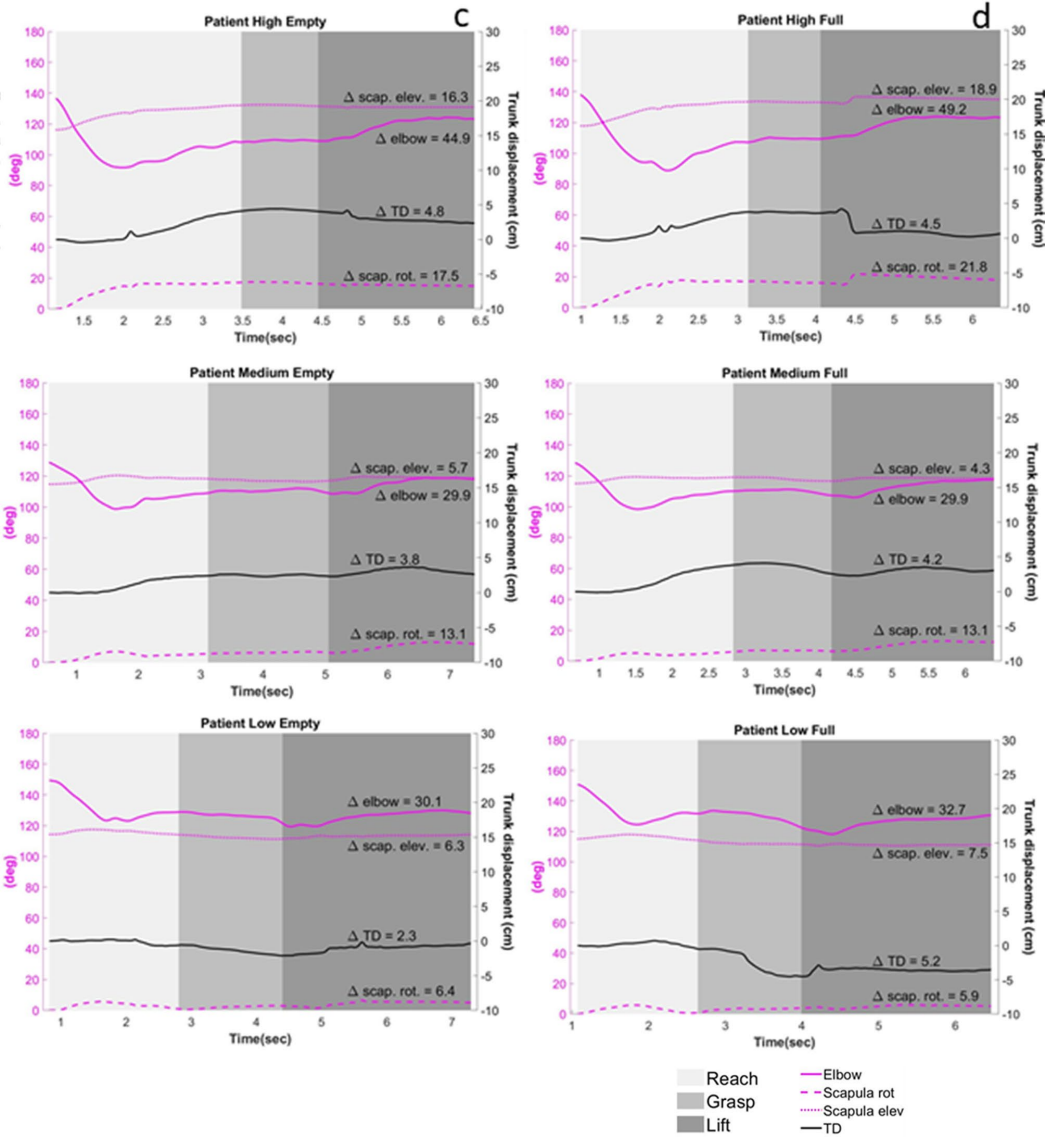
Graph of the RTG movement of one post-stroke participant (P29; FMA = 33; moderate-severe) and one healthy control (C01). The movement of the different joints in the three different heights and two different weights according to the three parts of the movement: Reach, Grasp, and Lift. *Left axis* (purple) represents movement in degrees of the scapula and elbow; *right axis* (black) represents TD in cm. Control’s RTG to the different heights with an empty cup [line (**a**)] and a full cup [line (**b**)]; Patient’s RTG to the differnt heights with an empty cup [line (**c**)] and a full cup [line (**d**)]. D: the difference in deg/cm form T1 to T4. As is clearly evident, the patient uses trunk displacement and scapular elevation as part of the movement, whereas the control participant hardly shows any involvement of the trun. Abbreviations: RTG: Reach to grasp; TD: Trunk displacement; scap: Scapula; cm: centimeters; deg: degrees.

Group effect: The FTR was significantly lower among participants with stroke compared to the control group for both phases (*Grasp:* F_1,770_ = 340.65, *p* < 0.001; *Lift:* F_1,745_ = 190, *p* < 0.001; see Fig. 4). As stroke severity increased, participants produced significantly less force per unit time (*Grasp:* F_3,781_ = 133, *p* < 0.001; *Lift:* F_3,741_ = 96.41, *p* < 0.001). Among the stroke group participants produced higher force per time unit when testing the non-dominant hand (*Grasp*: F_1,485_ = 19.18, *p* < 0.001; *Lift*: F_1,468_ = 57.67, *p* < 0.001), which was not dependent on stroke severity.

Height Effect: The *height* of the table had a significant effect on FTR (*Grasp:* F_2,504_ = 4.13, *p* = 0.017; *Lift:* F_2,490_ = 4.28, *p* = 0.014). More force was applied per unit time at the lowest table height for both groups. In the control group, during both phases, the FTR gradually decreased with increasing table height, with a significant difference between the low height and the highest height (*Grasp: p* = 0.022; *Lift*: *p* = 0.002). This difference was not found in the stroke group’s data. The results of the FTR are presented in Fig. S1 in the Supplementary*.*

Severity × weight interaction: FTR was significantly higher for a full cup compared to an empty one for both groups during both phases (*Grasp:* F_1,781_ = 8.52, *p* = 0.004, *Lift:* F_1,741_ = 18.57, *p* < 0.001), with the difference between a full and an empty cup being the largest for the control group, and decreased with increasing stroke severity (*Grasp:* F_3,741_ = 3.07, *p* = 0.027, *Lift:* F_3,781_ = 4.95, *p* = 0.002).

#### CV of force

Group effect: During the *Grasp* phase there was no significant difference in the CV of force between the two groups (F_3,792_ = 133, *p* < 0.001), however, during the *Lift* phase the CV of the stroke group was significantly higher than of the control (F_1,795_ = 5.97, *p* = 0.015). The moderate-severe group had significantly higher CV values compared to the mild and moderate severity groups and to the control group (F_3,786_ = 34.89, *p* < 0.001). During the *Grasp* phase, both groups had higher CV values when the non-dominant hand was tested (F_1,792_ = 12.83, *p* < 0.001), while in the *Lift* phase, this difference was evident only for the control group (F_1,282_ = 3.98, *p* = 0.047).

Height effect: *Grasp:* When grasping the cup at the lowest height the CV was significantly higher than when grasping the cup at the highest height (F_2,544_ = 3.802, *p* = 0.023), with no difference between the groups.

Weight effect: There was no effect of weight on the CV for either phase.

#### Number of force peaks (NFP)

Group effect: The participants with stroke had significantly higher NFP than the control participants during both phases (*Grasp*: F_1,745_ = 83.54, *p* < 0.001; *Lift*: F_1,766_ = 26.65, *p* < 0.001), and increased with increasing severity (*Grasp:* F_3,742_ = 43.69, *p* < 0.001; *Lift:* F_3,768_ = 42.81, *p* < 0.001). Both groups demonstrated higher NFP during the *Grasp* phase when testing the dominant hand (F_1,745_ = 18.45, *p* < 0.001). This difference was present during the *Lift* phase only for the stroke group (F_1,448_ = 13.87, *p* < 0.001).

Height effect: *Lift:* The NFP increased with increasing table height (F_2,536_ = 5.59, *p* = 0.04).

Weight effect: There was no effect of weight on the NFP for either phase. However, when analyzing the weight × severity interaction, the moderate-severe stroke group demonstrated the greatest effect of weight on the NFP, with higher NFP when lifting an empty cup (Empty: 6.4 ± 7.2; Full: 4.7 ± 4.1; F_3,768_ = 2.775, *p* = 0.04). The results of the NFP are presented in Fig. S2 in the Supplementary.

#### Kinematic analysis

Tables 3 and 4 summarize the results of the kinematic variables for the stroke and the control groups.

**Table 3:** Mean values of kinematic variables: group and lateralization effects.

	Group						Congruent									
	Reach		F	Lift		F	Reach				F	Lift				
	Stroke (n = 30)	Control (n = 16)		Stroke (n = 30)	Control (n = 16)		Stroke		Control			Stroke		Control		F
							Yes	No	Yes	No		Yes	No	Yes	No	
Mean velocity (mm/s)	**205 (4.9)**	**397.6 (6.6)**	**F**_**1,626**_ = 543.26***	**86.8 (1.9)**	**152.5 (2.5)**	**F**_**1,715**_ = 426.97***	**231.1 (5.8)**	**180.8 (5.8)**	**416.8 (10.2)**	**371.9 (13.1)**	**F**_**1,626**_ = 36.55***	**90.2 (2.2)**	**82.3 (2.2)**	**167 (3.8)**	**130.5 (4.9)**	**F**_**1,715**_ = 29.39***
Peak velocity (mm/s)	**468 (10.6)**	**770.6 (14.2)**	**F**_**1,587**_ = 290.6***	**243.8 (6.9)**	**484 (9.4)**	**F**_**1,583**_ = 145.63***	**506.6 (13.4)**	**437.6 (13.4)**	**803.9 (20.5)**	**724.1 (26.5)**	**F**_**1,587**_ = 18.26***	**252.2 (7.5)**	**228.3 (7.5)**	**409.5 (12.6)**	**305.3 (16.3)**	**F**_**1,583**_ = 19.1***
TTPV (s)	**2.2 (0.04)**	**1.4 (0.05)**	**F**_**1,691**_ = 144.26***	**7.8 (0.2)**	**3.2 (0.3)**	**F**_**1,723**_ = 130.05***	**2.1 (0.1)**	**2.4 (0.1)**	1.4 (0.04)	1.4 (0.05)	**F**_**1,691**_ = 7.17***	8.1 (0.4)	7.6 (0.4)	3.3 (0.1)	3.1 (0.1)	F_1,723_ = 0.33
Log (NJ)	**3.4 (0.02)**	**2.7 (0.03)**	**F**_**1,774**_ = 294.46***	**3.9 (0.03)**	**3.2 (0.04)**	**F**_**1,740**_ = 164.48***	**3.2(0.04)**	**3.6 (0.04)**	2.7(0.02)	2.7 (0.02)	**F**_**1,774**_ = 37.48***	4.0 (0.05)	3.9 (0.05)	3.3 (0.03)	3.3 (0.03)	F_1,740_ = 2.16
IC	**1.4 (0.01)**	**1.2 (0.02)**	**F**_**1,593**_ = 37.6***	**2.5 (0.1)**	**2.2 (0.1)**	**F**_**1,643**_ = 4.71*	**1.4 (0.02)**	**1.5 (0.02)**	**1.3 (0.01)**	**1.2 (0.01)**	**F**_**1,593**_ = 7.25**	**2.8 (0.1)**	**2.1 (0.1)**	**2.1 (0.06)**	**1.8 (0.07)**	**F**_**1,643**_ = 13.09***

Summary of the results. Group: lists the mean values (SE); bolded values denote a significant difference between the groups. Congruent: lists the mean values (SE); Yes—the examined hand was the dominant one; No—the examined hand was the non-dominant one. Asterisks denote the *p* value: *p ≤ 0.05, **p ≤ 0.01, ***p ≤ 0.001.

*mm* Millimeters, *s* Seconds, *TTPV* Time to-peak-velocity, *NJ* Normalized Jerk, *IC* Index of curvature.

**Table 4:** Mean values of kinematic variables: effect of height, weight and severity.

	Height		Weight		Severity	
	Reach	Lift	Reach	Lift	Reach	Lift
	F	F	F	F	F	F
Duration (s)	F_2,508_ = 1.50	**F**_**2,533**_ = 3.43*	F_1,768_ = 1.23	F_1,519_ = 0.09	**F**_**3,768**_ = 146.10***	**F**_**3,519**_ = 65.07***
Mean velocity (cm/s)	**F**_**3,602**_ = 111.96***	**F**_**2,603**_ = 10.53***	F_1,662_ = 0.48	F_1,693_ = 0.00	**F**_**3,662**_ = 286.39***	**F**_**3,693**_ = 183.57***
Peak velocity (cm/s)	**F**_**2,577**_ = 144.559***	**F**_**2,485**_ = 15.54***	F_1,663_ = 0.02	F_1,570_ = 1.49	**F**_**3,663**_ = 144.55**	**F**_**3,570**_ = 49.23**
TTPV (s)	**F**_**2,555**_ = 6.85***	F_2,559_ = 2.35	F_1,684_ = 3.80	F_1,744_ = 0.35	**F**_**3,684**_ = 77.25***	**F**_**3,744**_ = 88.55***
Log (NJ)	**F**_**2,511**_ = 3.08*	**F**_**2,570**_ = 3.38*	F_1,783_ = 1.26	F_1,740_ = 0.51	**F**_**3,783**_ = 177.79***	**F**_**3,740**_ = 123.64***
IC	**F**_**2,470**_ = 40.46***	F_2,269_ = 0.92	F_1666_ = 0.68	F_1,256_ = 0.08	**F**_**3,666**_ = 22.55***	**F**_**3,256**_ = 8.58***

Summary of the kinematic results. Bolded values denote a significant difference between the groups. Height, Weight, Severity: list the F value for the differences across this parameter; Asterisks denote the *p* value: *p ≤ 0.05, **p ≤ 0.01, ***p ≤ 0.001.

*mm* Millimeters, s Seconds, *TTPV* Time to-peak-velocity, *NJ* Normalized Jerk, *IC* Index of curvature.

#### Movement velocity

Mean and peak velocity results are presented in Fig. S3 in the Supplementary and summarized in Tables 3 and 4.

#### Smoothness of movement

*Normalized jerk (NJ)* Group Effect: The logNJ value was higher among participants with stroke compared to healthy controls for all phases of the task (*Reach*: F_1,808_ = 330.74, *p* < 0.001*; Grasp:* F_1,811_ = 228.04, *p* < 0.001; *Lift:* F_1,776_ = 161.26, *p* < 0.001; *Total task*: F_1,781_ = 245.1, *p* < 0.001). During the *Grasp* and *Lift* phases, logNJ values increased with increasing severity (F_3,276_ = 3.65, *p* = 0.013). Among the stroke group the logNJ was higher during the *Reach* phase when testing the non-dominant hand (F_1,487_ = 44.42, *p* < 0.001).

Height effect: The height had a significant effect on the log(NJ) during *Reach* (F_2,511_ = 3.08, *p* = 0.047), *Grasp* (F_2,532_ = 3.39, *p* = 0.034), and *Lift* (F_2,570_ = 3.38, *p* = 0.035), with a smoother movement when *reaching* and *grasping* at the higher height (*p* = 0.042), and a smoother movement when *lifting* at the lower height (*p* = 0.029). Weight Effect: The weight of the cup had no influence the log(NJ) values during either phase. The results of the logNJ are presented in Fig. S4 in the Supplementary.

*Index of curvature* (IC) The results of the IC are presented in Fig. S5 in the Supplementary.

#### Joint movement

TD, scapular movement and the movement of the elbow joint during the task are presented in Fig. 4.

*Trunk displacement (TD)* Participants with stroke demonstrated significantly greater displacement of the trunk compared to the control group (F_1,750_ = 89.65, *p* < 0.001)) (See Fig. S6 in the Supplementary). Both height and severity significantly affected TD. At the highest height, TD was greater compared to the lowest height (F_2,523_ = 50.46, *p* < 0.001) for both groups, and increased with increasing severity (F_3,753_ = 70.95, *p* < 0.001).

*Scapular elevation* Participants with stroke demonstrated higher D_angle_ scapular elevation values for all phases of the task (*Reach*: F_1,503_ = 6.77, *p* = 0.01; *Grasp*: F_1,481_ = 9.05, *p* = 0.003 *Lift*: F_1,554_ = 6.69, *p* = 0.01). At the higher height, both groups demonstrated significant higher D_angle_ scapular elevation compared to the lower height for all phases of the task (*Reach*: F_2,257_ = 25.73, *p* < 0.001; *Grasp*: F_2,257_ = 25.73, *p* < 0.001 *Lift:* F_2,302_ = 114.40, *p* < 0.001). LMM analysis demonstrated significant height × group interaction in the D_angle_ scapular elevation for *Reach* and *Lift* phases (*Reach*: F_2,301_ = 4.86, *p* = 0.008; *Lift*: F_2,302_ = 8.80, *p* < 0.001), meaning greater difference between stroke and control group at the higher height.

*Scapular rotation* Significant difference in D_angle_ scapular rotation between the two groups was evident during *Grasp* and *Lift* (*Grasp*: F_1,739_ = 108.39, *p* < 0.001; *Lift*: F_1,675_ = 15.36, *p* < 0.001). There was a significant difference in D_angle_ scapular rotation for all phases between the higher height and the lower height. Greater D_angle_ values were evident when *reaching* and *lifting* at the lower height (*Reach*: F_2,468_ = 89.54, *p* < 0.001; *Lift*: F_2,611_ = 30.21, *p* < 0.001), and greater D_angle_ values were evident when *grasping* at the higher height (F_2,603_ = 55.05, *p* < 0.001).

*Elbow* Participants with stroke demonstrated lower D_angle_ values of the elbow angle compared to the control group for the *Reach* and *Lift* phases (*Reach*: F_1,771_ = 30.40, *p* < 0.001; *Lift*: F_2,370_ = 134.53, *p* < 0.001), indicating they extended their elbow to a lesser extent while reaching and lifting. The maximal elbow extension angle (T1-T4) at the lower height was significantly larger than at the higher height (F_2,612_ = 430, *p* < 0.001).

#### Correlations

We found a significant negative correlation between the logNJ and the FTR in both phases (*Grasp*: Control: rs =  − 0.686, *p* < 0.001; Stroke: rs =  − 0.922, *p* < 0.001; *Lift*: Control: rs =  − 0.576, *p* < 0.001; Stroke: rs =  − 0.842, *p* < 0.001). That is, when participants applied less force per unit time, the movement was jerkier. This correlation was very strong for the stroke group and moderate for the control group.

In the stroke group we found a significant strong correlation between the logNJ and the NFP during both the *Grasp* phase (rs = 0.663, *p* < 0.001) and the *Lift* phase (rs = 0.778, *p* < 0.001). This relationship was not present in the control group’s data.

A significant negative correlation was found in the stroke group’s data between the FTR and the NFP, which was strong during the *Grasp* phase (rs =  − 0.662, *p* < 0.001), and moderate during the *Lift* phase (rs =  − 0.581, *p* < 0.001). This relationship was not found for the control group.

A moderate negative relationship was found between TD and the maximal angle of the elbow (Control: rs =  − 0.525, *p* < 0.001; Stroke: rs =  − 0.417, *p* < 0.001), indicating that when participants used more trunk displacement, they used less elbow extension.

In the stroke group we found a significant correlation between the TD and the NFP which was moderate during *Grasp* (rs = 0.478, *p* < 0.001) and strong during the *Lift* phase (rs = 0.605, *p* < 0.001). In addition, a moderate correlation was found between the TD and the logNJ during the *Lift* phase (rs = 0.549, *p* < 0.001).

## Discussion

We compared the effect of an object’s weight and height on the kinematics and on the force regulation of 46 individuals—30 persons with stroke and 16 age-matched healthy control participants—engaged in a functional task: they reached for, grasped, and lifted a cup, which was either empty or full of water (and thus of two different weights), and located at one of three different heights. To the best of our knowledge, this is the first study to examine how the combination of different heights and different weights of an object in a functional RTG task affects the kinematics, the force regulation, and the quality of movement of persons with stroke compared to healthy individuals. Target height affected both force calibration and kinematics, while target weight affected only the force calibration. We found a significant difference between the two groups in all velocity measures, trajectory smoothness, and straightness. Participants with stroke used excessive trunk displacement and scapular elevation to compensate for lack of elbow extension, which was more pronounced in the higher height compared to the lower one, and more pronounced with increasing stroke severity. We found no difference between the two groups in the mean and peak forces they produced. Force regulation of stroke participants was less efficient, and more variable compared to the control group. For stroke participants, the mean force, the peak force and FTR were higher when the lesion side was the non-dominant one. In accordance with our fourth hypothesis, the differences in kinematics and in force regulation were more pronounced as the severity of the stroke impairment was higher.

### Force regulation

Improvements in grasp-force regulation were found to be associated with functional recovery of the affected arm post-stroke^70,71^, and therefore should be an integral component of the rehabilitation plan. One of our main goals was to understand how force regulation varies with object weight and height, and with impairment level post-stroke, as it is directly correlated to the ability to perform ADL tasks^14,29,30^. In the current study, we used a cup with an embedded force sensor during a functional task of reach, grasp, and lift in order to resemble, as closely as possible, a common component of ADL.

Previous studies that examined the difference between stroke participants and healthy controls in their mean^30,72^ and peak forces^11,73^, reported variable results. Some of them reported higher peak forces among persons with stroke^74^ whereas some reported lower mean^30^ and peak forces^75^ or no difference^76^. The difference in the findings between different studies could derive from differences in the examined tasks.

The results of the current study emphasize that describing the mean- and the peak-force values are not sufficient in order to understand the motor behavior of persons with stroke. Variability analysis, lateralization, and a distinction between severity levels within the stroke group gives a more nuanced understanding of their motor control.

Interestingly, we found that, unlike healthy participants, participants with stroke did not scale their force application according to the demands of the task. This was more pronounced with increasing stroke severity. The difficulty of participants with stroke in force scaling was also reported by Parry, et al.^77^ where 12 post-stroke participants were asked to perform different grasping and lifting tasks.

Both height and weight were found to influence most of the force-control variables. We found that higher mean force is used when the table is lower, and the weight is heavier. While the use of more force to lift a heavier weight is expected as a means to prevent the cup from slipping, the finding regarding the height effect is surprising, as one might think that more force should be applied when reaching for a higher-up object, as it is supposed to be a more difficult height to lift to. This could be explained by the force–velocity relationship of a muscle, which is a is a fundamental principle of skeletal muscle physiology. The slower a skeletal muscle shortens the greater the force it can generate during contraction and vice versa^78^. During high reach participants produced higher velocity and generated less force.

Force regulation of persons with stroke is inefficient and inconsistent. This affects their independence level^79^. In order to demonstrate the variability and inefficiency of force regulation, we used three measures: CV^29,30,59^; Force-to-time ratio; and NFP. CV is a common variable, that was tested in previous studies^29,30^. We added the NFP and the force-to-time ratio analyses to provide a more comprehensive examination, and to offer new insights regarding the regulation of force when post-stroke individuals perform a functional task. We posit that a single variability measure does not sufficiently capture the intricacies of force regulation by individuals with stroke, but examining the combination of these three measures provides a complementary understanding of how force regulation is altered in stroke.

Similar to Lindberg et al.^29^, we found that the difference in CV varied with the task. A significant difference in the CV value was not found between the two groups during the *Grasp* phase; however, CV was higher for the stroke group when *lifting* the cup. We suggest that the CV does not sufficiently reflect the variability in planning and execution of the grasp-and-lift movement. Therefore, we also examined another measure that reflects the variability of force control: the number of force peaks (NFP). This measure was not examined for force analysis in previous studies. However, number of peaks, as a measure of movement smoothness, is commonly used in movement-velocity and acceleration analysis^47,60–62,80^. In the context of force regulation, this measure may reflect the efficiency of planning and execution of the grasp-and-lift movement. High NFP reflects inefficient and inconsistent force regulation, and the tendency of persons with stroke to intermittently apply too much and too little force on the grasped object. Accordingly, we found a significantly higher NFP value in the movements of the stroke group compared to those of the control group, and a higher NFP value with increasing stroke severity.

The force-to-time ratio reflects the efficiency of force regulation over time, by calculating how much force is applied per time unit. In addition, it reflects the fatigue post-stroke individuals may experience by producing the same amount of mean force compared to healthy individuals, but over an extended period of time, which may lead to fatigue of their UL. Unlike the CV, which was affected to a limited extent by the weight or the height of the cup, the FTR was affected by both the weight and the height of the grasped object. The two force regulation measures were found to be negatively correlated: when less force was applied per time unit, the number of force peaks for the same movement phase was higher. These findings were also related to the laterality of the lesion; Higher *FTR* values were evident when the stroke affected their non-dominant hand, while higher *NFP* values were present when the stroke affected their dominant hand.

These results highlight the force-control characteristics of RTG ability post-stroke. It can be carefully assumed that healthy individuals anticipate and scale the force needed to apply before lifting a cup, as opposed to post-stroke participants, who produced less force per unit of time, which suggests an altered anticipation and preparation for lifting. This interpretation is supported by Nowak et al.^81^, who assert that when known objects are grasped and lifted, predictive force control is used, and by Frenkel-Toledo et al.^82^, who reported decreased task-specific adaptability among stroke participants. Our study has shown that this ability is altered with increasing stroke severity.

### Kinematics

Our study confirmed previous findings that during reaching, persons with stroke tend to demonstrate longer movement time^24,38^, as well as lower mean^8^ and peak velocity and a later time-to-peak-velocity^5,19,43^ compared with healthy controls. Our results show that even participants with mild impairment demonstrated altered peak velocity and difficulty during acceleration.

We found that for both groups, the higher the target, the higher was their mean velocity. The increasing velocity along with the increased distance can be explained by the isochrony principle, which captures the empirical observation that the duration of movements involved in the generation of motion paths with similar geometrical forms but with different lengths are nearly equal^83^, meaning that movement duration is nearly independent of movement size^60,83^.

Participants with stroke demonstrated a jerkier trajectory during all phases of the task. These results are consistent with previous studies which assessed smoothness either by NJ^84^ or by the number of motor-units^85^ and found that UL motion of persons with stroke was characterized by less smooth hand paths. The novelty of the current study is the effect of height and weight on this measure. The movement was jerkier when reaching and lifting a higher target and the NJ increased with increasing severity. This was also correlated with more compensations performed in the higher height, as reaching and lifting to a higher target is a harder task, involving a greater effort.

IC was used to assess trajectory straightness. Previous studies have found that IC of persons with stroke tends to be higher during unilateral reaching^5,21^, while no difference in IC was found between the affected and the non-affected arm during bilateral reaching^20^. In the current study, the trajectory of participants with stroke was less straight than that of the control group, during both reach and lift. It is interesting to note that while control participants demonstrated a straighter trajectory while lifting the cup at a low height [compared to the highest height] (similar to the mild-impaired group), the moderate and moderate-severe groups tended to have a more curved path during low lifting, perhaps due to difficulty in recruiting the anterior deltoid muscle and controlling the wrist and fingers while grasping. This result could be explained by the additional recruitment of the lateral deltoid in reaching task of people with stroke as was reported by McCrea et al. ^86^.

The weight of the cup did not have a significant effect on either NJ or IC. These results may be explained by the findings of Nowak^14^ showing that an object's weight imposes constraints mainly on the shaping of grasp aperture, positioning of the fingers on the object and force control, and less on proximal joints, which may contribute more to trajectory straightness and smoothness. However, as IC values during lifting were higher than the values during reaching, it might be that the condition of carrying as opposed to not carrying a weight affected the IC, but the difference between the two weight conditions (either empty-273 g or full cup-443 g) was not sufficiently large to affect the NJ or the IC.

### Quality of movement

The contribution of the trunk movement to reaching, which we report here, is consistent with previous literature describing TD as a common compensatory motor behavior during reaching^20,54,87^.

When examining the effect of the height of the table on the ranges of motion of participants with stroke, most of our hypotheses were confirmed. Participants with stroke demonstrated an increased range of TD, scapular elevation, scapular rotation as the height of the table was higher. These results are consistent with a previous study that found in persons with stroke, the deficit in selectively using combinations of trunk stabilizing muscles was more evident for upward reaching than for downward reaching^41^. When reaching the lower height, participants with stroke managed to produce more elbow extension with less TD and scapular elevation; That is, they generated a more selective movement with less compensations.

These results contrast with those of Valdes et al. and Reisman et al.^20,88^, who reported that individuals with stroke exhibited larger values of trunk flexion^20^ and reduced inter-joint coordination and trunk control^88^ when downward reaching to a target at knee height in-front of them. Our study presents a more comprehensive characterization of functional reaching to an object at three different heights. The difference in findings might derive from the difference in the experimental setup: the table in our study was located parallel to the thigh, in the “low” height, rather than in front of the participant, and we used an actual object in the RTG task, rather than a moving^20^ or a fixed^88^ handle.

Based on the results we present here, we suggest that rehabilitation programs focusing on UL function post-stroke should consider the height of the target, making the rehabilitation progress more gradual, from a low height, enabling the performance of more selective movements even for more severely impaired post-stroke participants, and progressing to higher heights of the reached target, thereby minimizing compensatory strategies, which are undesirable in the long term, by that enabling a more selective and efficient movement.

It is important to note that not all our results were consistent with our hypotheses. We found no effect of weight on kinematic performance, regardless of height or impairment level. This result might suggest that participants with stroke (who were told whether the cup was empty or full) maintain to some extent their ability to anticipate the weight of the object and adapt their movement accordingly, but not their force control. As mentioned previously, this lack of effect of the weight on kinematics may be a factor of the weights used; However, this is the weight of a drinking cup in a RTG task.

### Effect of lesion side (dominant vs. non-dominant)

The surprising finding that the mean and peak forces of stroke participants as well as the FTR were higher when the affected arm was their non-dominant one suggests that this difference may derive from the affected cortical brain side. The participants in our study suffered from hemispheric stroke lesion, which involved brain structures supplied by the MCA, ACA and PCA. Hermsdörfer et al.^11^ reported that either affected hand demonstrated an impaired force control. Their conclusion was that in persons with cortical stroke grip forces may be massively increased, and the safety margins may be excessive. Interestingly, our results show that when the non-dominant hand was used, *too much* force was recruited for completing the task, while when the dominant hand was used, *too little* force was recruited for task completion, independent of stroke severity. According to the model of Mani et al.^89^, which is based on kinematic measures, the left hemisphere is responsible for predicting and accounting for limb dynamics and the right hemisphere for stabilizing limb position through impedance control mechanisms. It could be that when the dominant side of the brain is affected, it impedes the ability to anticipate and to adapt the forces according to the requirements of the task, while when the non-dominant hemisphere is affected, it impedes the ability of the grasping hand to stabilize the object, which leads to producing significantly higher mean and peak forces. The current results extend the applicability of Mani's model to include the effect of the cortical hemisphere not only on kinematics, but also on force regulation.

### Clinical implications

Results of this study extend previous findings by investigating motor control and force production during upward and downward functional reaching. An increased understanding of underlying motor control processes would facilitate therapists in development and implementation of effective interventions to improve motor skills in individuals with stroke and to build a gradual rehabilitation program for improving function of the arm, taking into consideration variables like the weight of the object and the target height, according to the patient's impairment level, as well as the laterality of the lesion.

The data from this study were used to build an algorithm to detect altered forces and compensatory movements^55^, to be ultimately used for post-stroke game-based robotic technology^90^.

### Main contribution

The main contribution of this paper is the integration of both force-control analysis and movement analysis in a *functional* reach-to-grasp task, with changing task environment and a built-in measurement tool.

We found that the height of the reached target affects both force calibration and kinematics, while its weight affects only the force calibration when post-stroke and healthy individuals perform a reach-to-grasp task.

### Study limitations

Dividing the participants with stroke into three severity levels resulted in relatively small subgroups, specifically of the moderately-severe impairment group. While it is a precious group to include in the analysis, it is prohibitively difficult to recruit a large group of participants into it who can complete the tested task. This has clinical implications as well: it is important for clinicians to understand how motor control varies with severity.

Since grip force was measured without a gyroscope sensor, we were not able to measure the orientation of the cup during the lifting phase, but only the grip force applied to it.

Finally, we examined only the contralateral hand and not both hands, which limit our ability to conclude on hemispheric specificity for movement control mechanism. The analysis was based on hospital records, which included CT scans; we did not independently image the brain of the participants, and no MRI lesion data were assessed; future studies would benefit from collection and analysis of these data as well, to give a fuller understanding of the contributing factors.

## Supplementary Information

Supplementary Information.

## Data Availability

The datasets used and/or analyzed during the current study are available from the corresponding author on reasonable request.

## References

[1] Waller SM (2016). Impaired motor preparation and execution during standing reach in people with chronic stroke. Neurosci. Lett..

[2] Rathore SS, Hinn AR, Cooper LS, Tyroler HA, Rosamond WD (2002). Characterization of incident stroke signs and symptoms: findings from the atherosclerosis risk in communities study. Stroke.

[3] Baniña MC, Mullick AA, McFadyen BJ, Levin MF (2017). Upper limb obstacle avoidance behavior in individuals with stroke. Neurorehabilit. Neural Repair.

[4] Hatem SM (2016). Rehabilitation of motor function after stroke: A multiple systematic review focused on techniques to stimulate upper extremity recovery. Front. Hum. Neurosci..

[5] Cirstea M, Levin MF (2000). Compensatory strategies for reaching in stroke. Brain.

[6] Alaverdashvili M, Whishaw IQ (2013). A behavioral method for identifying recovery and compensation: Hand use in a preclinical stroke model using the single pellet reaching task. J. Neurosci. Biobehav. Rev..

[7] Veerbeek JM, Langbroek-Amersfoort AC, Van Wegen EE, Meskers CG, Kwakkel G (2017). Effects of robot-assisted therapy for the upper limb after stroke: A systematic review and meta-analysis. Neurorehabilit. Neural Repair.

[8] Kim C-Y (2015). Effect of spatial target reaching training based on visual biofeedback on the upper extremity function of hemiplegic stroke patients. J. Phys. Ther. Sci..

[9] van Vliet P, Pelton TA, Hollands KL, Carey L, Wing AM (2013). Neuroscience findings on coordination of reaching to grasp an object: implications for research. Neurorehabilit. Neural Repair.

[10] Zaal FT, Bootsma R, van Wieringen PC (1998). Coordination in prehension Information-based coupling of reaching and grasping. Exp. Brain Res..

[11] Hermsdörfer J, Hagl E, Nowak D, Marquardt CJCN (2003). Grip force control during object manipulation in cerebral stroke. Clin. Neurophysiol..

[12] Flanagan JR, Wing AM (1997). The role of internal models in motion planning and control: evidence from grip force adjustments during movements of hand-held loads. J. Neurosci..

[13] Pilon J-F, De Serres SJ, Feldman AG (2007). Threshold position control of arm movement with anticipatory increase in grip force. Exp. Brain Res..

[14] Nowak DA (2008). The impact of stroke on the performance of grasping: Usefulness of kinetic and kinematic motion analysis. Neurosci. Biobehav. Rev..

[15] Ameli M, Dafotakis M, Fink GR, Nowak DA (2008). Predictive force programming in the grip-lift task: The role of memory links between arbitrary cues and object weight. Neuropsychologia.

[16] Flash T, Hogan N (1985). The coordination of arm movements: an experimentally confirmed mathematical model. J. Neurosci..

[17] Levin MF (1996). Interjoint coordination during pointing movements is disrupted in spastic hemiparesis. Brain.

[18] Levy-Tzedek S, Hanassy S, Abboud S, Maidenbaum S, Amedi A (2012). Fast, accurate reaching movements with a visual-to-auditory sensory substitution device. Restor. Neurol. Neurosci..

[19] Shaikh T, Goussev V, Feldman AG, Levin MF (2014). Arm–trunk coordination for beyond-the-reach movements in adults with stroke. Neurorehabilit. Neural Repair.

[20] Valdés BA, Glegg SM, Van der Loos HM (2017). Trunk compensation during bimanual reaching at different heights by healthy and hemiparetic adults. J. Mot. Behav..

[21] Michaelsen SM, Luta A, Roby-Brami A, Levin MF (2001). Effect of trunk restraint on the recovery of reaching movements in hemiparetic patients. Stroke.

[22] Michaelsen SM, Dannenbaum R, Levin MF (2006). Task-specific training with trunk restraint on arm recovery in stroke: randomized control trial. Stroke.

[23] Merdler T, Liebermann DG, Levin MF, Berman S (2013). Arm-plane representation of shoulder compensation during pointing movements in patients with stroke. J. Electromyogr. Kinesiol..

[24] Murphy MA, Willén C, Sunnerhagen KS (2011). Kinematic variables quantifying upper-extremity performance after stroke during reaching and drinking from a glass. Neurorehabilit. Neural Repair.

[25] Osu R (2011). Quantifying the quality of hand movement in stroke patients through three-dimensional curvature. J. NeuroEng. Rehabilit..

[26] Thrane G, Sunnerhagen KS, Murphy MA (2020). Upper limb kinematics during the first year after stroke: The stroke arm longitudinal study at the University of Gothenburg (SALGOT). J. NeuroEng. Rehabilit..

[27] Lang CE, Wagner JM, Edwards DF, Sahrmann SA, Dromerick AW (2006). Recovery of grasp versus reach in people with hemiparesis poststroke. Neurorehabilit. Neural Repair.

[28] Enders LR, Seo NJ (2015). Altered phalanx force direction during power grip following stroke. Exp. Brain Res..

[29] Lindberg PG (2012). Affected and unaffected quantitative aspects of grip force control in hemiparetic patients after stroke. Brain Res..

[30] Ye Y (2014). Kinetic measurements of hand motor impairments after mild to moderate stroke using grip control tasks. J. NeuroEng. Rehabilit..

[31] Blennerhassett JM, Carey LM, Matyas TA (2008). Clinical measures of handgrip limitation relate to impaired pinch grip force control after stroke. J. Hand Ther..

[32] Scharoun SM, Gonzalez DA, Roy EA, Bryden PJ (2018). End-state comfort across the lifespan: A cross-sectional investigation of how movement context influences motor planning in an overturned glass task. Mot. Control.

[33] Rosenbaum DA (2017). Knowing Hands: The Cognitive Psychology of Manual Control.

[34] Rosenbaum DA, Chapman KM, Weigelt M, Weiss DJ, van der Wel R (2012). Cognition, action, and object manipulation. Psychol. Bull..

[35] Rosenbaum DA, Sauerberger KS (2019). End-state comfort meets pre-crastination. Psychol. Res..

[36] Rosenbaum DA (2009). The posture-based motion planning framework: new findings related to object
manipulation, moving around obstacles, moving in three spatial dimensions, and haptic tracking. Progress in Motor
Control.

[37] van Dokkum L (2014). The contribution of kinematics in the assessment of upper limb motor recovery early after stroke. Neurorehabilit. Neural Repair.

[38] Stewart JC, Gordon J, Winstein CJ (2014). Control of reach extent with the paretic and nonparetic arms after unilateral sensorimotor stroke: Kinematic differences based on side of brain damage. Exp. Brain Res..

[39] Stewart JC, Gordon J, Winstein CJ (2014). Control of reach extent with the paretic and nonparetic arms after unilateral sensorimotor stroke II: Planning and adjustments to control movement distance. Exp. Brain Res..

[40] Wagner JM, Rhodes JA, Patten CJCB (2008). Reproducibility and minimal detectable change of three-dimensional kinematic analysis of reaching tasks in people with hemiparesis after stroke. Phys. Ther..

[41] Gera G, McGlade KE, Reisman DS, Scholz JP (2016). Trunk muscle coordination during upward and downward reaching in stroke survivors. Mot. Control.

[42] Park M (2010). The comparison of muscle activation on low-reaching and high-reaching in patient with stroke. J. Phys. Ther. Sci..

[43] van Vliet PM, Sheridan MR (2009). Ability to adjust reach extent in the hemiplegic arm. Physiotherapy.

[44] Michaelsen SM, Jacobs S, Roby-Brami A, Levin MF (2004). Compensation for distal impairments of grasping in adults with hemiparesis. Exp. Brain Res..

[45] Blennerhassett JM, Matyas TA, Carey LM (2007). Impaired discrimination of surface friction contributes to pinch grip deficit after stroke. Neurorehabilit. Neural Repair.

[46] Blennerhassett JM, Carey LM, Matyas TA (2006). Grip force regulation during pinch grip lifts under somatosensory guidance: Comparison between people with stroke and healthy controls. Arch. Phys. Med. Rehabilit..

[47] Liebermann DG, Berman S, Weiss PL, Levin MF (2012). Kinematics of reaching movements in a 2-D virtual environment in adults with and without stroke. IEEE Trans. Neural Syst. Rehabilit. Eng..

[48] Kamper DG, McKenna-Cole AN, Kahn LE, Reinkensmeyer DJ (2002). Alterations in reaching after stroke and their relation to movement direction and impairment severity. Arch. Phys. Med. Rehabilit..

[49] Demers M, Levin MF (2020). Kinematic validity of reaching in a 2D virtual environment for arm rehabilitation after stroke. IEEE Trans. Neural Syst. Rehabilit. Eng..

[50] Park H, Kim S, Winstein CJ, Gordon J, Schweighofer N (2016). Short-duration and intensive training improves long-term reaching performance in individuals with chronic stroke. Neurorehabilit. Neural Repair.

[51] Anderson C, Rajamani K, Pardo V, Adamo DE (2018). Asymmetries in force matching are related to side of stroke in right-handed individuals. Neurosci. Lett..

[52] Woytowicz EJ (2017). Determining levels of upper extremity movement impairment by applying a cluster analysis to the Fugl-Meyer assessment of the upper extremity in chronic stroke. Arch. Phys. Med. Rehabilit..

[53] Aprile I (2014). Kinematic analysis of the upper limb motor strategies in stroke patients as a tool towards advanced neurorehabilitation strategies: a preliminary study. BioMed Res. Int..

[54] Levin MF, Liebermann DG, Parmet Y, Berman S (2016). Compensatory versus noncompensatory shoulder movements used for reaching in stroke. Neurorehabilit. Neural Repair.

[55] Kashi S, Feingold Polak R, Lerner B, Rokach L, Levy-Tzedek S (2020). A machine-learning model for automatic detection of movement compensations in stroke patients. IEEE Trans. Emerg. Top. Comput..

[56] World-Health-Organization. International classification of functioning. 28–66 (2001).

[57] Quintas R (2012). Describing functioning, disability, and health with the international classification of functioning, disability, and health brief core set for stroke. Am. J. Phys. Med. Rehabilit..

[58] Aparicio P, Diedrichsen J, Ivry RB (2005). Effects of focal basal ganglia lesions on timing and force control. Brain Cogn..

[59] Lodha N, Naik SK, Coombes SA, Cauraugh JH (2010). Force control and degree of motor impairments in chronic stroke. Clin. Neurophysiol..

[60] Levy-Tzedek S, Tov MB, Karniel A (2011). Rhythmic movements are larger and faster but with the same frequency on removal of visual feedback. J. Neurophysiol..

[61] Levy-Tzedek S, Krebs HI, Song D, Hogan N, Poizner H (2010). Non-monotonicity on a spatio-temporally defined cyclic task: Evidence of two movement types?. Exp. Brain Res..

[62] Yaffe JA, Zlotnik Y, Ifergane G, Levy-Tzedek S (2020). Implicit task switching in Parkinson’s disease is preserved when on medication. PLoS ONE.

[63] Levy-Tzedek S (2017). Changes in predictive task switching with age and with cognitive load. Front. Aging Neurosci..

[64] Murphy MA, Baniña MC, Levin MF (2017). Perceptuo-motor planning during functional reaching after stroke. Exp. Brain Res..

[65] Roby-Brami A, Jacobs S, Bennis N, Levin MF (2003). Hand orientation for grasping and arm joint rotation patterns in healthy subjects and hemiparetic stroke patients. Brain Res..

[66] Rohrer B (2002). Movement smoothness changes during stroke recovery. J. Neurosci..

[67] Hogan N, Sternad D (2009). Sensitivity of smoothness measures to movement duration, amplitude, and arrests. J. Mot. Behav..

[68] Buma FE (2016). Brain activation is related to smoothness of upper limb movements after stroke. Exp. Brain Res..

[69] Kwakkel G (2019). Standardized measurement of quality of upper limb movement after stroke: Consensus-based core recommendations from the Second Stroke Recovery and Rehabilitation Roundtable. Neurorehabilitation Neural Repair.

[70] Harris JE, Eng JJJS (2010). Strength training improves upper-limb function in individuals with stroke: A meta-analysis. Stroke.

[71] McDonnell MN, Hillier SL, Ridding MC, Miles TS (2006). Impairments in precision grip correlate with functional measures in adult hemiplegia. Clin. Neurophysiol..

[72] Kurihara J, Lee B, Hara D, Noguchi N, Yamazaki T (2019). Increased center of pressure trajectory of the finger during precision grip task in stroke patients. Exp. Brain Res..

[73] Ding Q, Patten CJCB (2018). External biomechanical constraints impair maximal voluntary grip force stability post-stroke. Clin. Biomech..

[74] Nowak DA, Hermsdörfer J, Topka H (2003). Deficits of predictive grip force control during object manipulation in acute stroke. J. Neurol..

[75] Aruin AS (2005). Support-specific modulation of grip force in individuals with hemiparesis. Arch. Phys. Med. Rehabilit..

[76] Schaefer SY, DeJong SL, Cherry KM, Lang CE (2012). Grip type and task goal modify reach-to-grasp performance in post-stroke hemiparesis. Mot. Control.

[77] Parry R (2019). Effects of hand configuration on the grasping, holding, and placement of an instrumented object in patients with hemiparesis. Front. Neurol..

[78] Alcazar J, Csapo R, Ara I, Alegre LM (2019). On the shape of the force-velocity relationship in skeletal muscles: The linear, the hyperbolic, and the double-hyperbolic. Front. Physiol..

[79] Boissy P, Bourbonnais D, Carlotti MM, Gravel D, Arsenault BA (1999). Maximal grip force in chronic stroke subjects and its relationship to global upper extremity function. Clin. Rehabil..

[80] Levy-Tzedek S (2017). Motor errors lead to enhanced performance in older adults. Sci. Rep..

[81] Nowak DA, Glasauer S, Hermsdörfer J (2013). Force control in object manipulation—A model for the study of sensorimotor control strategies. Neurosci. Biobehav. Rev..

[82] Frenkel-Toledo S, Yamanaka J, Friedman J, Feldman AG, Levin MF (2019). Referent control of anticipatory grip force during reaching in stroke: An experimental and modeling study. Exp. Brain Res..

[83] Bennequin D, Fuchs R, Berthoz A, Flash T (2009). Movement timing and invariance arise from several geometries. PLoS Comput. Biol..

[84] Liebermann, D. G., Levin, M. F., McIntyre, J., Weiss, P. L. & Berman, S. in *Annual International Conference of the IEEE Engineering in Medicine and Biology* 5242–5245 (IEEE, 2010). 10.1109/IEMBS.2010.5626297 21096047

[85] Tomita Y, Mullick AA, Levin MF (2018). Reduced kinematic redundancy and motor equivalence during whole-body reaching in individuals with chronic stroke. Neurorehabilit. Neural Repair.

[86] McCrea PH, Eng JJ, Hodgson AJ (2005). Saturated muscle activation contributes to compensatory reaching strategies after stroke. J. Neurophysiol..

[87] Massie CL, Malcolm MP, Greene DP, Browning RC (2012). Kinematic motion analysis and muscle activation patterns of continuous reaching in survivors of stroke. J. Mot. Behav..

[88] Reisman DS, Scholz JP (2006). Workspace location influences joint coordination during reaching in post-stroke hemiparesis. Exp. Brain Res..

[89] Mani S (2013). Contralesional motor deficits after unilateral stroke reflect hemisphere-specific control mechanisms. Brain.

[90] Feingold Polak, R. & Levy-Tzedek, S. in *Proceedings of the 2020 ACM/IEEE International Conference on Human–Robot Interaction* 151–160 (2020).

