# Supplementary material for: The effects of an object’s height and weight on force calibration and kinematics when post-stroke and healthy individuals reach and grasp
Source: Sci Rep. 2021 Oct 18;11:20559. doi: 10.1038/s41598-021-00036-9 (PMC8523696; doi:10.1038/s41598-021-00036-9)
Supplement: Supplementary file 1 — Supplementary Information. [file 41598_2021_36_MOESM1_ESM.pdf]

The effects of an object's height and weight on force calibration and kinematics when post-stroke and healthy individuals reach and grasp

Ronit Feingold-Polak, MScPT[1]†, Anna Yelkin, MScPT[1,2]†, Shmil Edelman, MME[3], Amir Shapiro, PhD.[3], Shelly Levy-Tzedek, PhD[1,4,5*]

†These authors contributed equally to this work

1. Recanati School for Community Health Professions, Department of Physical Therapy, Ben-Gurion University of the Negev, Beer-Sheva, Israel

2. Beit Hadar Rehabilitation Center, Ashdod, Israel

3. Department of Mechanical Engineering, Ben-Gurion University of the Negev, Beer-Sheva, Israel

4. Zlotowski Center for Neuroscience, Ben-Gurion University of the Negev, Beer-Sheva, Israel

5. Freiburg Institute for Advanced Studies (FRIAS), University of Freiburg, Freiburg, Germany

* Corresponding author: shelly@bgu.ac.il, 972-8-6479560

Ben-Gurion Blvd, Beer-Sheva, Israel

Table S1: Demographic characteristics of the participants

Participant No.	Age (years)	Gender (M/F)	Time post-stroke (days)	Lesion type (I/H)	Lesion location	Affected side (R/L) (recorded side for control)	Dominant hand tested (Y/N)	FMA score/66	Chedoke score/49
**Stroke**									
P01	58	M	38	I	MCA	R	Y	56	46
P02	56	M	91	I	MCA	R	Y	36	23
P03	85	F	38	H	MCA	L	N	44	34
P04	62	M	51	I	ACA	L	Y	38	28
P05	80	F	64	I	BG	L	N	48	33
P06	74	F	52	I	MCA	L	N	56	42
P07	55	F	60	I	MCA	L	N	50	41
P08	81	F	26	I	MCA+BG	L	N	46	33
P09	67	M	18	I	MCA+BG	L	N	59	46
P10	79	F	39	I	Cerebellum	R	Y	49	34
P11	82	F	28	I	MCA	L	N	50	38
P12	75	F	29	I	PCA	R	Y	57	45
P13	78	F	42	I	MCA	R	Y	52	41
P14	66	F	38	I	MCA	R	Y	58	44
P15	63	M	34	I	Pons	R	Y	57	45
P16	63	M	71	H	BG	R	Y	33	16
P17	59	M	37	I	MCA	R	Y	35	32
P18	71	M	33	I	MCA	L	N	42	30
P19	59	F	62	I	MCA	R	Y	40	31
P20	59	M	97	H	FL	L	N	30	19
P21	74	M	37	I	BG	L	N	53	42
P22	71	F	27	I	MCA	L	N	62	47
P23	83	M	34	I	MCA	L	Y	33	27
P24	79	M	40	I	MCA	R	Y	59	45
P25	60	F	29	I	MCA	L	N	46	38
P26	80	M	30	I	PCA	R	Y	54	43
P27	64	M	32	I	PCA	L	N	35	19
P28	79	M	76	I	MCA	R	Y	30	22
P29	78	M	56	H	BG	L	N	33	29
P30	70	F	72	I	MCA	R	Y	55	35
**Mean ± SD**	70.33±9.3	M=53.3% F=46.7%	46.03±19.9	I=86.6% H=13.4%		R=46.6% L=53.4%	Y=53.3% N=46.7%	46.5±10.1	34.9±9.1
**Range**	55-85							30-62	16-47
**Healthy Controls**									
C01	51	F	NA	NA	NA	R	Y	NA	NA
C02	59	F	NA	NA	NA	L	N	NA	NA
C03	85	M	NA	NA	NA	L	N	NA	NA
C04	82	F	NA	NA	NA	R	N	NA	NA
C05	61	F	NA	NA	NA	L	N	NA	NA
C06	66	M	NA	NA	NA	L	N	NA	NA
C07	79	F	NA	NA	NA	R	Y	NA	NA
C08	85	M	NA	NA	NA	R	Y	NA	NA
C09	76	F	NA	NA	NA	R	Y	NA	NA
C10	79	F	NA	NA	NA	R	Y	NA	NA
C11	63	F	NA	NA	NA	R	Y	NA	NA

C12	70	F	NA	NA	NA	R	Y	NA	NA
C13	63	F	NA	NA	NA	R	Y	NA	NA
C14	52	F	NA	NA	NA	L	N	NA	NA
C15	57	M	NA	NA	NA	L	Y	NA	NA
C16	78	M	NA	NA	NA	L	N	NA	NA
**Mean ± SD**	69.1±11.6	M=31.2% F=68.8%				R=56.2% L=43.8%	Y=56.2% N=43.8%		
**range**	51-85								

Abbreviations: P-Patients, C-Control; M-male; F-female; I-ischemic; H-hemorrhagic; MCA-Middle cerebral artery; ACA-Anterior cerebral artery; BG-Basal ganglia; PCA-Posterior cerebral artery; FL-Frontal lobe; NA-Not Applicable; R-right; L-left; Y-yes; N-no; FMA-Fugl-Meyer Assessment; SD-standard deviation.

Time point calculations

In order to analyse the data, the movement was divided into three logical phase: Reaching for the cup, Grasp and Lift. The segments were determined using the following four time points: movement initiation (T1), the time at which the cup was grasped (T2), the time at which the cup was lifted (T3), and the end of the movement, once the cup was placed on the shelf (T4)[39]. The combinations of the time points produced the phases of movement: Phase 1 "Reach to grasp" (T1 to T2: from starting position until grasp of the cup), Phase 2 "Grasp" (T2 to T3), Phase 3 "Lift" (T3 to T4: from grasp until placing the cup on the shelf), and the whole movement (T1 to T4). First T4 was calculated, then T3, T2 and finally T1. They were calculated as follows: *Movement initiation (T1):* The speed trace of the wrist movement measured by the wrist sensor was scanned from the start of the recording to T2, when the cup was grasped. T1 was the time at which the wrist speed exceeded 10% of its maximal value during this phase.

- *Cup grasp (T2):* The maximal value of the force within the first 100 data points on the force trace was calculated ($F_{baseline\_max}$). T2 was the time at which the force trace first reached a value of 0.2 N beyond $F_{baseline\_max}$.
- *Cup lift (T3):* The trace of the vertical position of the cup was scanned from the start of the recording to T4, and the maximum value was calculated – this was the highest cup location during the movement as measured by the Y component of the rigid body sensor (Y_{max}). The minimal cup height (Y_{min}) was similarly calculated. The vertical distance travelled by the cup $Y_{range} = Y_{max} - Y_{min}$ was then computed, and T3 was taken to be the time at which the cup reached a height equal to 10% of Y_{range} (above Y_{min}).
- *End of movement (T4):* The first 100 data points on the force trace were averaged, to establish the average baseline force value, and this value was subtracted from the force trace, to remove measurement noise. Then, the maximum value of the force in that movement was calculated (F_{max}). The last time point where the force value was 20% of F_{max} was identified, and the force trace was scanned from that point forward to find the first instance where the force value exceeded 5% of F_{max}. This point was determined as T4.

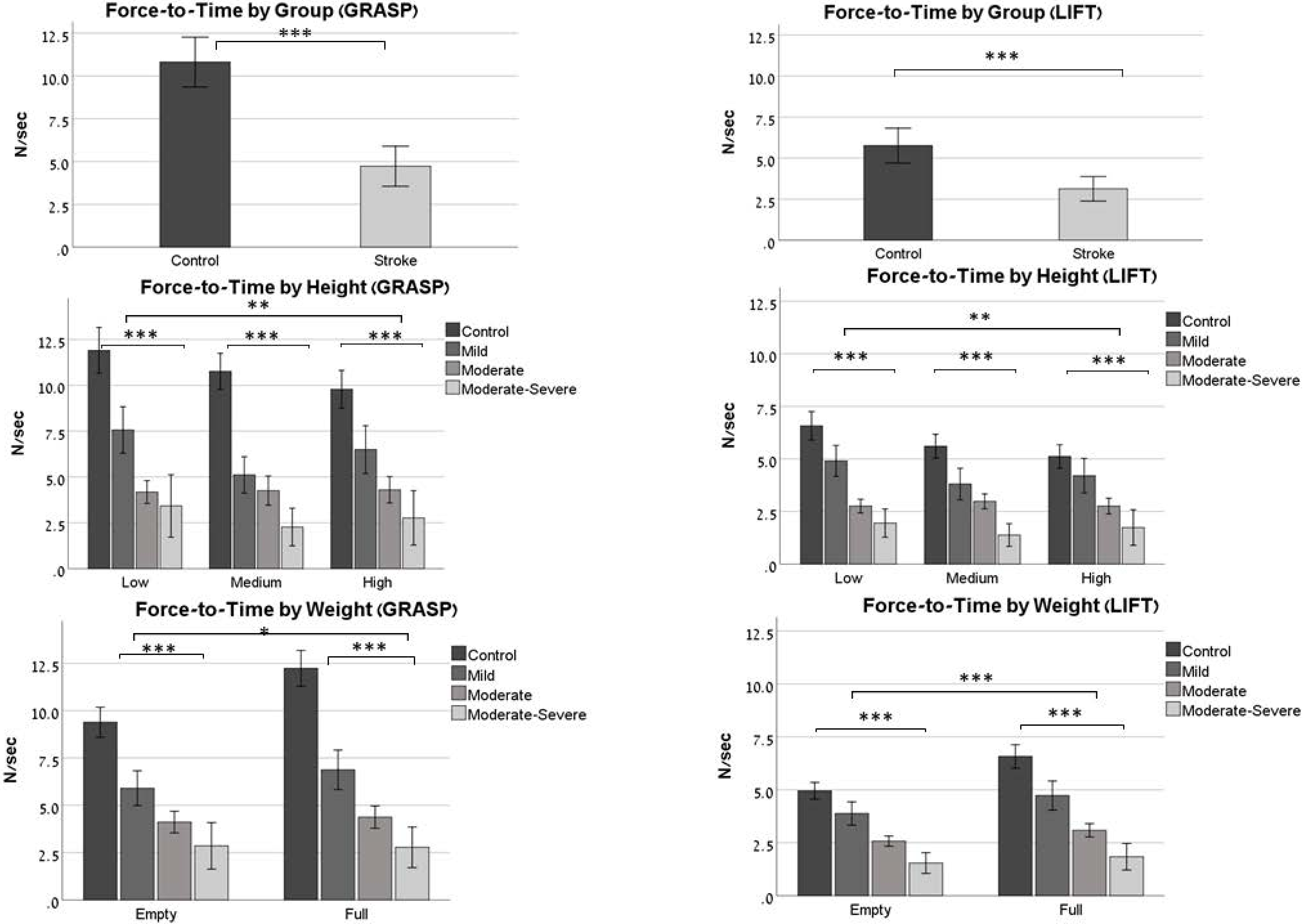

Force-to-Time by Group (GRASP)
N/sec
12.5
10.0
7.5
5.0
2.5
.0
Control
Stroke
***
Force-to-Time by Group (LIFT)
N/sec
12.5
10.0
7.5
5.0
2.5
.0
Control
Stroke
***
Force-to-Time by Height (GRASP)
N/sec
12.5
10.0
7.5
5.0
2.5
.0
Low
Medium
High
**
***
***
***
Control
Mild
Moderate
Moderate-Severe
Force-to-Time by Height (LIFT)
N/sec
12.5
10.0
7.5
5.0
2.5
.0
Low
Medium
High
**
***
***
***
Control
Mild
Moderate
Moderate-Severe
Force-to-Time by Weight (GRASP)
N/sec
12.5
10.0
7.5
5.0
2.5
.0
Empty
Full
*
***
***
Control
Mild
Moderate
Moderate-Severe
Force-to-Time by Weight (LIFT)
N/sec
12.5
10.0
7.5
5.0
2.5
.0
Empty
Full
***
***
***
Control
Mild
Moderate
Moderate-Severe

Figure S1. Force-to-time ratio (FTR; N/sec). FTR values for the Grasp phase (*left*), and the Lift phase (*right*). The top row shows the results by group (control/stroke). The middle row shows the results by height (low/medium/high), and by sub-group (control/mild impairment/moderate impairment/moderate-severe impairment). The bottom row shows the results by weight (empty/full cup, corresponding to light/heavy weight), and by sub-group. Asterisks denote the *p*-value: *$p \leq 0.05$, **$p \leq 0.01$, ***$p \leq 0.001$. Abbreviations: N-Newtons; sec-seconds; NS-not significant

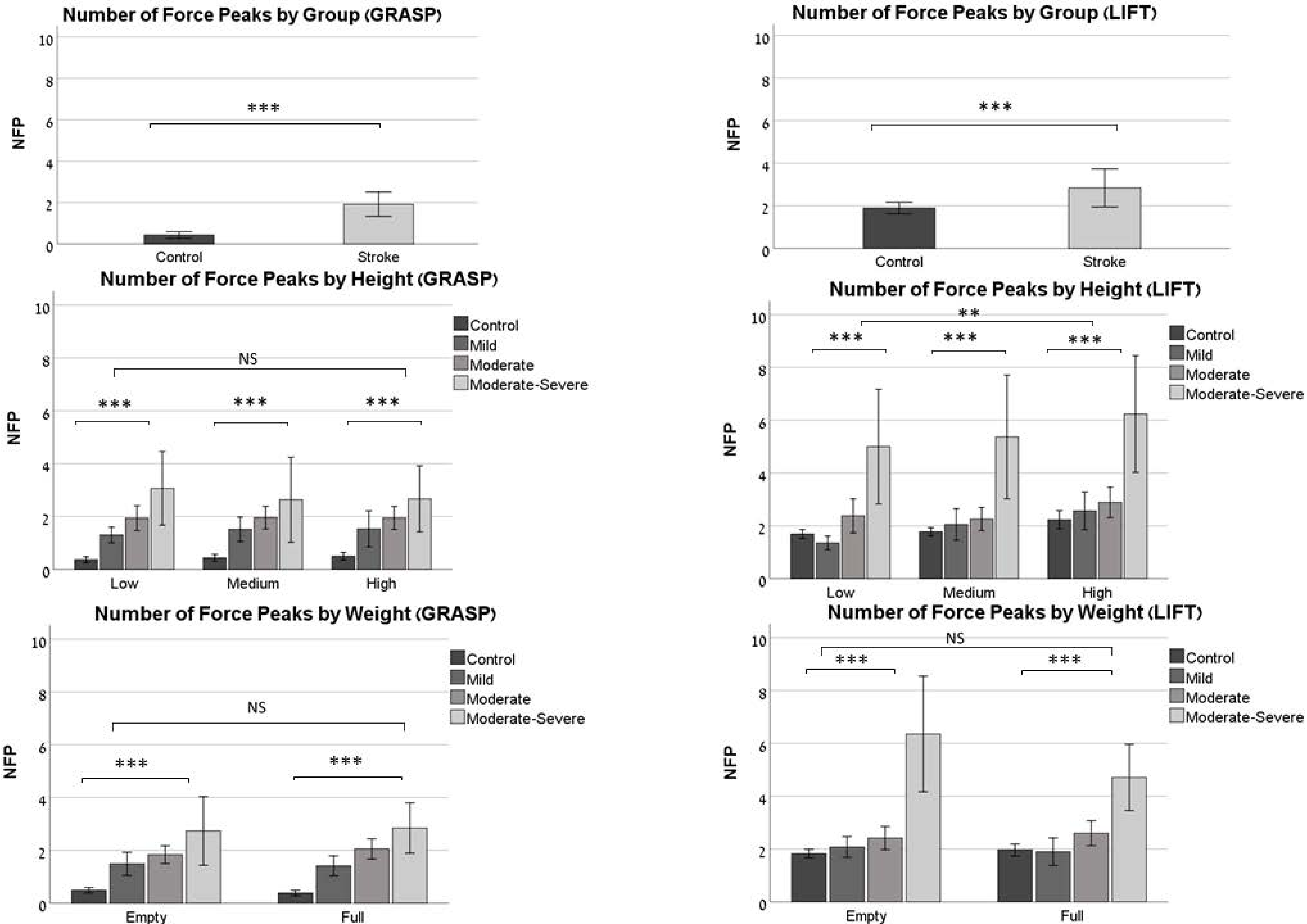
Number of Force Peaks by Group (GRASP)
NFP
0
2
4
6
8
10
***
Control
Stroke
Number of Force Peaks by Group (LIFT)
NFP
0
2
4
6
8
10
***
Control
Stroke
Number of Force Peaks by Height (GRASP)
NFP
0
2
4
6
8
10
Control
Mild
Moderate
Moderate-Severe
NS
***
***
***
Low
Medium
High
Number of Force Peaks by Height (LIFT)
NFP
0
2
4
6
8
10
Control
Mild
Moderate
Moderate-Severe
**
***
***
***
Low
Medium
High
Number of Force Peaks by Weight (GRASP)
NFP
0
2
4
6
8
10
Control
Mild
Moderate
Moderate-Severe
NS
***
***
Empty
Full
Number of Force Peaks by Weight (LIFT)
NFP
0
2
4
6
8
10
Control
Mild
Moderate
Moderate-Severe
NS
***
***
Empty
Full

Figure S2. Number of Force Peaks (NFP). NFP values for the Grasp phase (*left*), and the Lift phase (*right*). The top row shows the results by group (control/stroke). The middle row shows the results by height (low/medium/high), and by sub-group (control/mild impairment/moderate impairment/moderate-severe impairment). The bottom row shows the results by weight (empty/full cup, corresponding to light/heavy weight), and by sub-group. Asterisks denote the p-value: *$p \leq 0.05$, **$p \leq 0.01$, ***$p \leq 0.001$. Abbreviations: NS-not significant.

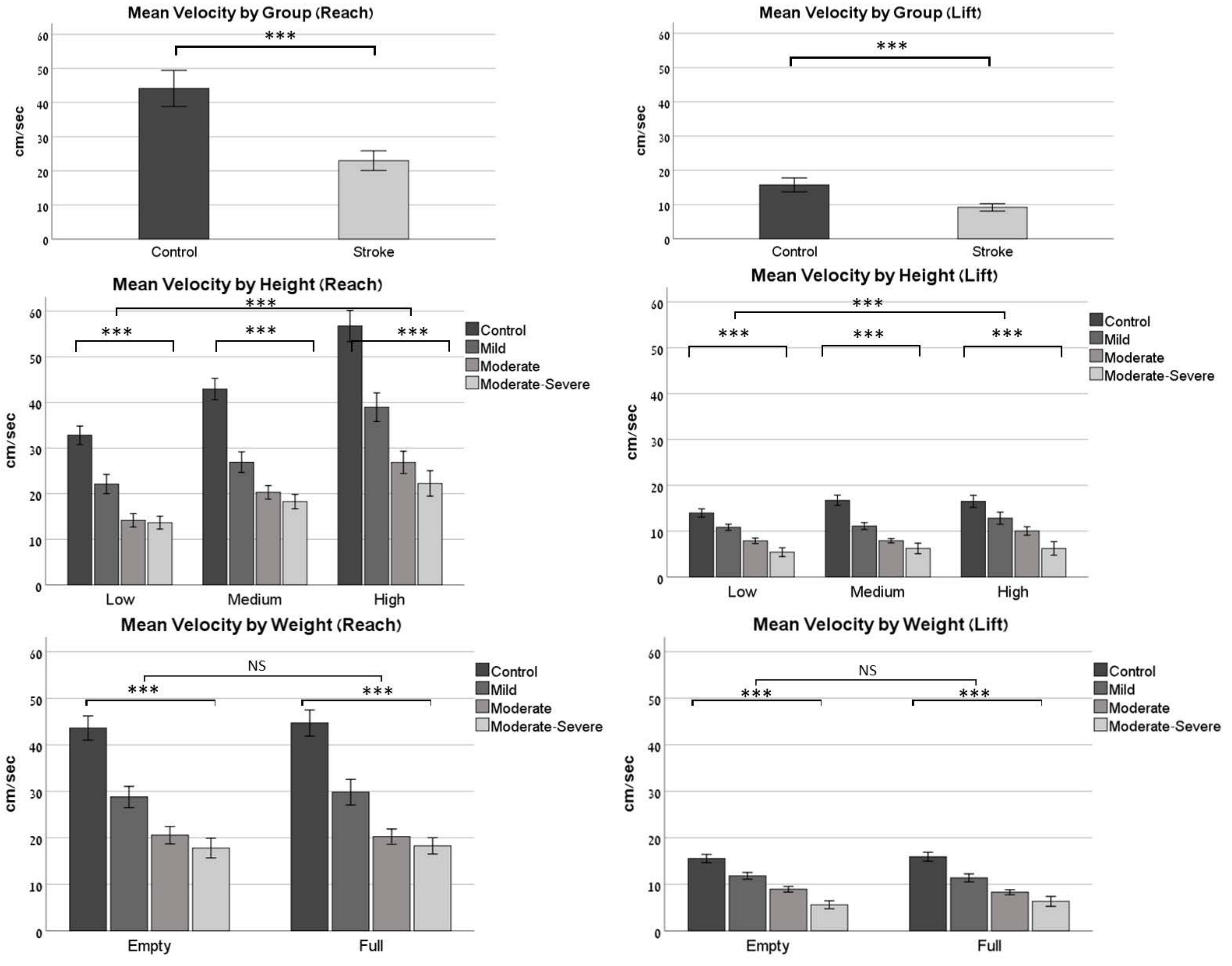

Mean Velocity by Group (Reach)
cm/sec
60
50
40
30
20
10
0
***
Control
Stroke
Mean Velocity by Group (Lift)
cm/sec
60
50
40
30
20
10
0
***
Control
Stroke
Mean Velocity by Height (Reach)
cm/sec
60
50
40
30
20
10
0
***
***
***
***
Control
Mild
Moderate
Moderate-Severe
Low
Medium
High
Mean Velocity by Height (Lift)
cm/sec
60
50
40
30
20
10
0
***
***
***
***
Control
Mild
Moderate
Moderate-Severe
Low
Medium
High
Mean Velocity by Weight (Reach)
cm/sec
60
50
40
30
20
10
0
NS
***
***
Control
Mild
Moderate
Moderate-Severe
Empty
Full
Mean Velocity by Weight (Lift)
cm/sec
60
50
40
30
20
10
0
NS
***
***
Control
Mild
Moderate
Moderate-Severe
Empty
Full

Figure S3. Mean Velocity. Mean velocity values for the Reach phase (*left*), and the Lift phase (*right*). The top row shows the results by group (control/stroke). The middle row shows the results by height (low/medium/high), and by sub-group (control/mild impairment/moderate impairment/moderate-severe impairment). The bottom row shows the results by weight (empty/full cup, corresponding to light/heavy weight), and by sub-group. Asterisks denote the *p*-value: **p*≤0.05, ***p*≤0.01, ****p*≤0.001. Abbreviations: cm-centimeter; sec-seconds.

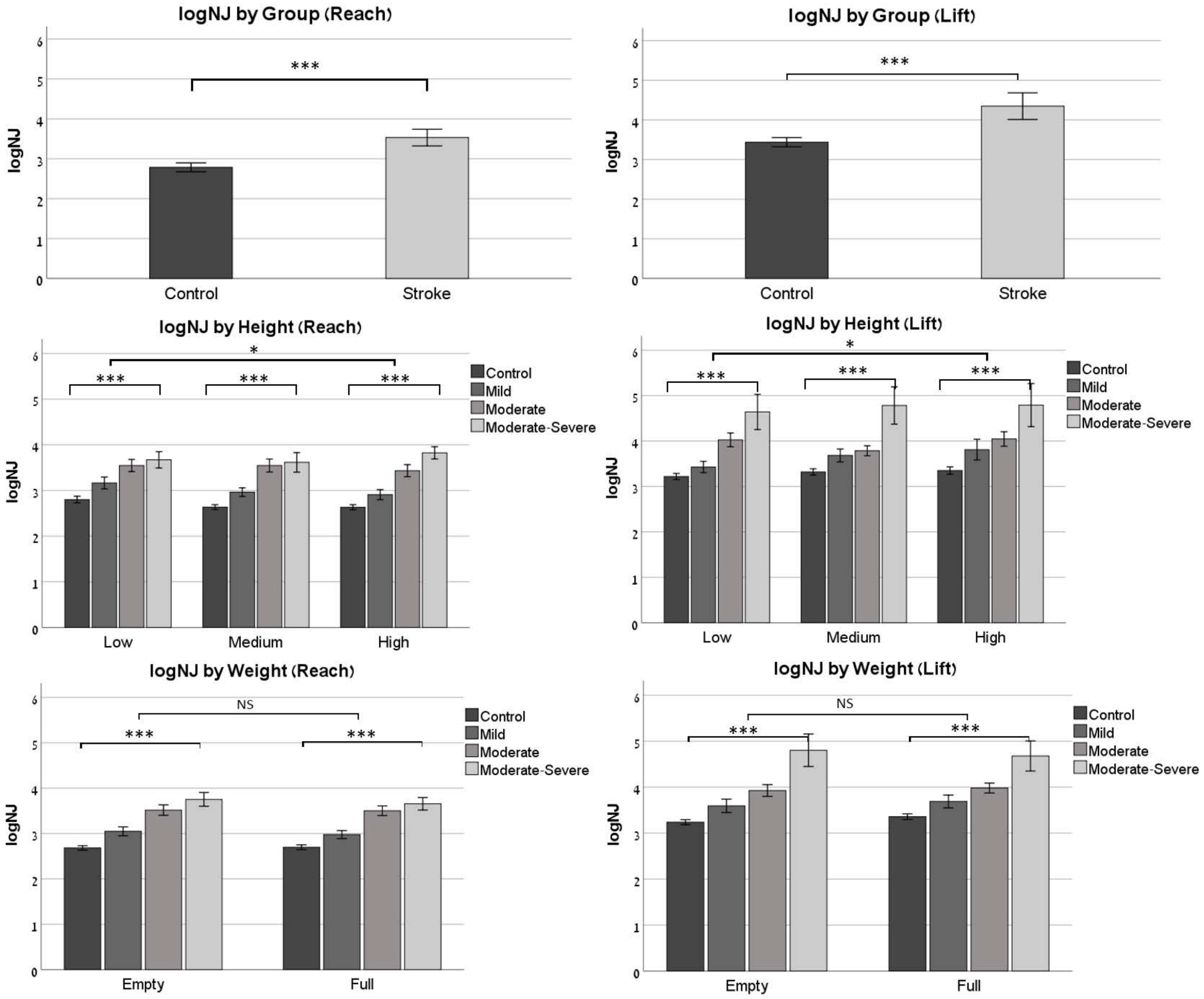

logNJ by Group (Reach)
logNJ
Control
Stroke
***
logNJ by Group (Lift)
logNJ
Control
Stroke
***
logNJ by Height (Reach)
logNJ
Low
Medium
High
*
***
Control
Mild
Moderate
Moderate-Severe
logNJ by Height (Lift)
logNJ
Low
Medium
High
*
***
Control
Mild
Moderate
Moderate-Severe
logNJ by Weight (Reach)
logNJ
Empty
Full
NS
***
Control
Mild
Moderate
Moderate-Severe
logNJ by Weight (Lift)
logNJ
Empty
Full
NS
***
Control
Mild
Moderate
Moderate-Severe

Figure S4. Normalized jerk (NJ). NJ values for the Reach phase (*left*), and the Lift phase (*right*). The top row shows the results by group (control/stroke). The middle row shows the results by height (low/medium/high), and by sub-group (control/mild impairment/moderate impairment/moderate-severe impairment). The bottom row shows the results by weight (empty/full cup, corresponding to light/heavy weight), and by sub-group. Asterisks denote the *p*-value: *$p \leq 0.05$, **$p \leq 0.01$, ***$p \leq 0.001$Abbreviations: NS-not significant.

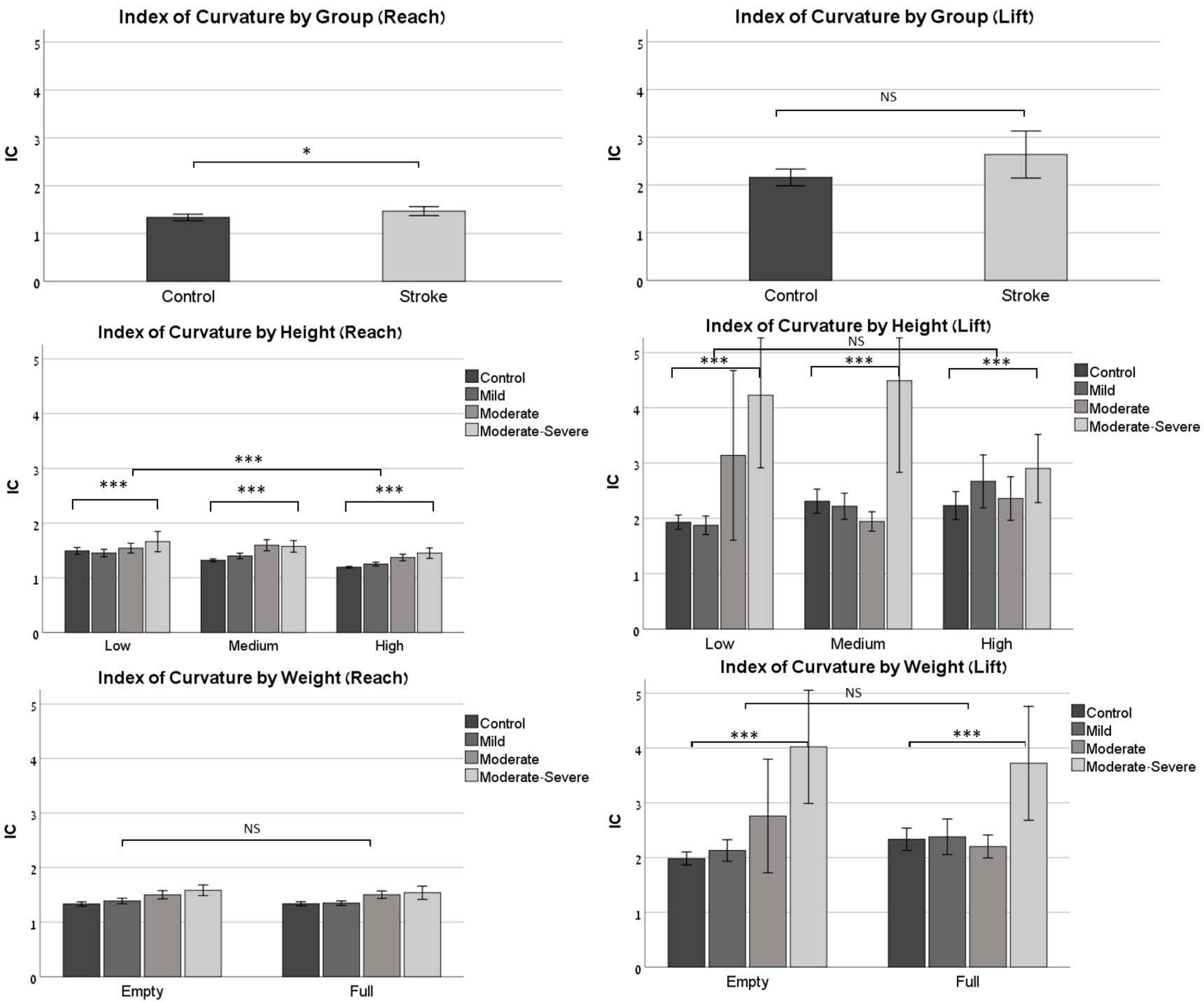

Index of Curvature by Group (Reach)
Index of Curvature by Group (Lift)
IC
*
NS
Control
Stroke
Index of Curvature by Height (Reach)
Index of Curvature by Height (Lift)
***
NS
Control
Mild
Moderate
Moderate-Severe
Low
Medium
High
Index of Curvature by Weight (Reach)
Index of Curvature by Weight (Lift)
Empty
Full

Figure S5. Index of Curvature (IC). IC values for the Reach phase (*left*), and the Lift phase (*right*). The top row shows the results by group (control/stroke). The middle row shows the results by height (low/medium/high), and by sub-group (control/mild impairment/moderate impairment/moderate-severe impairment). The bottom row shows the results by weight (empty/full cup, corresponding to light/heavy weight), and by sub-group. Asterisks denote the *p*-value: **p*≤0.05, ***p*≤0.01, ****p*≤0.001Abbreviations: NS-not significant.

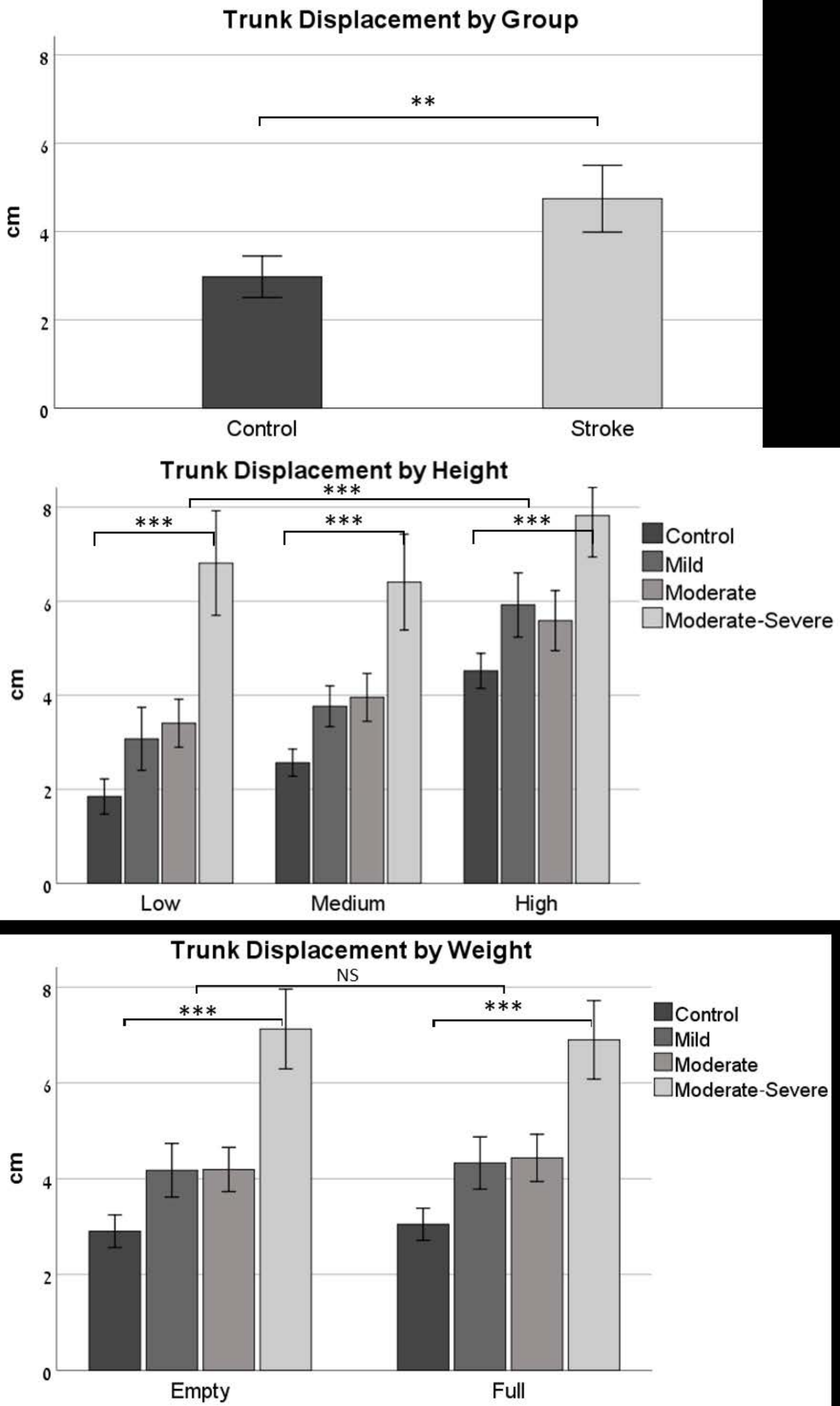

Trunk Displacement by Group
cm
0
2
4
6
8
**
Control
Stroke
Trunk Displacement by Height
cm
0
2
4
6
8
***
***
***
***
Low
Medium
High
Control
Mild
Moderate
Moderate-Severe
Trunk Displacement by Weight
cm
0
2
4
6
8
NS
***
***
Empty
Full
Control
Mild
Moderate
Moderate-Severe

Figure S6. Trunk Displacement (TD). TD represents the change of the trunk position in centimeters from T1 to T4. The top row shows the results by group (control/stroke). The middle row shows the results by height (low/medium/high), and by sub-group (control/mild impairment/moderate impairment/moderate-severe impairment). The bottom row shows the results by weight (empty/full cup, corresponding to light/heavy weight), and by sub-group. Asterisks denote the *p*-value: *$p \leq 0.05$, **$p \leq 0.01$, ***$p \leq 0.001$Abbreviations: cm-centimeters.
